# Analytical Solutions for Stochastic Vibration of Orthotropic Membrane under Random Impact Load

**DOI:** 10.3390/ma11071231

**Published:** 2018-07-18

**Authors:** Dong Li, Zhou-Lian Zheng, Rui Yang, Peng Zhang

**Affiliations:** 1 Scholl of Civil Engineering, Chongqing University, Chongqing 400045, China; 20151601001@cqu.edu.cn (D.L.); 201716021052@cqu.edu.cn (R.Y.); 201716131196@cqu.edu.cn (P.Z.); 2 Key Laboratory of New Technology for Construction of Cities in Mountain Area (Chongqing University), Ministry of Education, Chongqing 400045, China; 3 Chongqing Jianzhu College, Chongqing 400072, China

**Keywords:** orthotropic membrane, random impact load, stochastic vibration, geometric nonlinearity, FPK method

## Abstract

Orthotropic membrane materials have been applied in the numerous fields, such as civil engineering, space and aeronautics, and mechanical engineering, among others. During their serving lifespan, these membranes are always facing strong stochastic vibrations induced by the random impact load such as hail, heavy rain, and noise, among others. In this paper, the stochastic vibration problem of orthotropic membrane subjected to random impact load is investigated. The statistical characteristics of random impact load are initially obtained based on the stochastic pulse theory. Then, the Von Karman theory is applied to model the nonlinear vibration of membrane with geometric nonlinearity, which is then used to derive and solve the corresponding fokker–plank–kolmogorov (FPK). The theoretical model developed is validated by means of experiment study and monte carlo simulation (MCS) analysis. The effects of variables like pretension force, velocity of impact load, and material features on stochastic dynamic behavior of membranes are discussed in detail. This exposition provides theoretical framework for stochastic vibration control and design of membranes subjected to random dynamic load.

## 1. Introduction

Orthotropic membranes are lightweight, flexible structural elements used in numerous applications in construction building, space and aeronautics, and mechanical engineering, among others [1,2]. However, because of their specific aspects of lightweight and feeble stiffness, membranes are quite sensitive to impact load such as pulse wind, hails, rainstorm, and so on [3]. As a result, the impact load could cause the membrane to vibrate severely with large deformation, which may bring about structural failure [4]. Thus, it is necessary to study the vibration problem of membrane subjected to impact load.

In recent decades, a number of investigations on dynamic response of membrane under impact load have been performed. York et al. [5] applied the material point method to partition membrane surface, and solved the nonlinear vibration problem of membrane under impact load by the Lagrangian and Eulerian method. Porwal [6] performed the experiment of 2D membrane imposed by the ballistic impact load. The results obtained are valuable and useful for the design of fibrous materials such as body armor. Malla and Gionet [7] imagined a type of membrane planned to be a lunar habitat. Then, the analytical model of membrane under impact load was purposed and expected to apply in space colonization. Mostofi et al. [8] analyzed the fully clamped thin membrane subjected to impulsive load. The model could predict the structural dynamic response including large deflection result. Seide [9] dealt with the center normal deflection problem of uniformly loaded membrane by applying the expanded deflection function and an iterative method. The results will be helpful for the design of a sensor. Steinmann et al. [10] found the solution of mechanical problem of orthotropic sensor membranes under external pressure using Hermite polynomials and the Ritz method. Liu et al. [11] developed the theoretical and numerical model of rectangular orthotropic membrane, and studied the nonlinear vibration problem. Different from the study of scholars above, Zheng et al. [12] studied the stochastic vibration of membrane with the consideration of the stochastic characteristic of impact load. The statistical results including mean value, variance value, and mean square value were presented. Li et al. [13] assumed the impact load with the Normal distribution and investigated the stochastic vibration of membrane with the perturbation method. The analytical solution was validated by the experimental results and provided the suggestion of design of membrane.

From the open literature summarized above, it can be found that present studies of dynamic response of membrane under impact load are mainly confined to determined vibration analysis. However, the impact load in nature, such as pulse wind, hails, and explosion, among others, generally possesses uncertain characteristics [14], which will lead to the stochastic vibration with larger deformation for membrane. In this case, the actual deformation of membrane can exceed the prediction of analytical results based on the deterministic vibration theory, and increase their risk of collapse in use. Nevertheless, the present analytical model could not provide a reliable solution from probabilistic and statistical viewpoints. Therefore, it is worth purposing the stochastic vibration model of membrane subjected to uncertain impact load.

In this paper, the stochastic vibration problem of orthotropic rectangular membrane under impact load is investigated. First, the statistical aspects of impact load are determined based on the stochastic pulse theory. Then, the fokker–plank–kolmogorov (FPK) motion equation of membrane under impact load is established and solved, combining the Von Karman’s large deformation theory and FPK method. Consequently, a series of statistical results including probability density function (PDF) and cumulative distribution function (CDF) of displacement, mean value, and variance value of displacement are obtained. Furthermore, the theoretical model is validated by the experiment using the monte carlo simulation (MCS) method. Finally, the effects of pretension force, velocity of impact load, and membrane material on stochastic vibration behavior are discussed.

## 2. Theoretical Study

In this section, firstly, the stochastic vibration model of membrane under stochastic impact load will be outlined. Then, the differential motion equations and corresponding FPK equations will be derived and solved. Finally, the statistical characteristics of the dynamic problem of membrane can be determined.

### 2.1. Model Description

**Figure 1:**
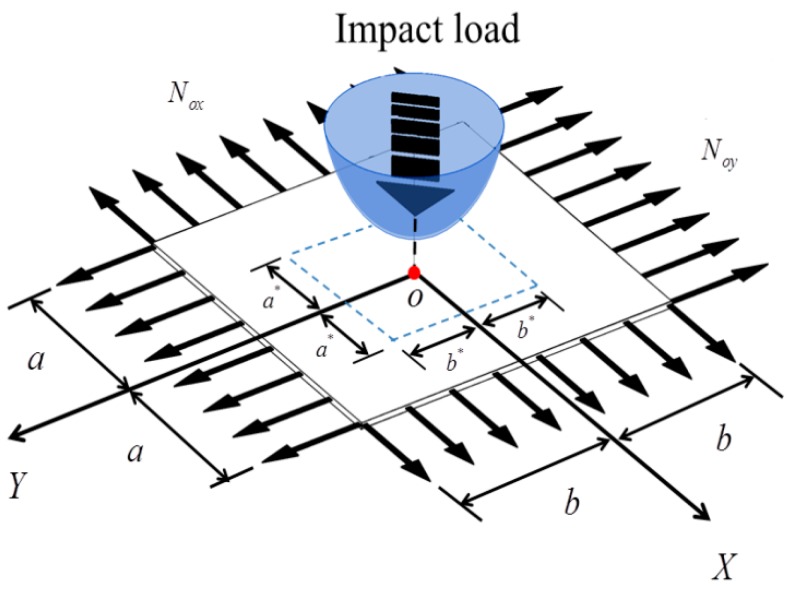
The model of orthotropic pre-stressed rectangular membrane under impact load.

Consider a homogeneous and orthotropic pre-stressed rectangular membrane with length 2a, width 2b, and thickness h, as is shown in Figure 1.The pretension force along the directions of x and y is $N_{0x}$ and $N_{0y}$, respectively.

The basic assumptions of membrane subjected to random impact load are as followings:

(1) Both membrane and impact load are symmetric. Furthermore, the center of impact load coincides with the center of membrane, namely (0,0);
(2) The impact load is the mass of homogeneous intensity $\rho^{*}$ and area with the length of $2a^{*}$ and the width of $2b^{*}$;
(3) The space distribution of impact load is symmetric and non-uniform;
(4) According to the central limit theorem, the amplitude of impact load follows a Gaussian distribution;
(5) The applied area of impact load can be defined as $\Omega_{0}=\left\{\left(x,y\right)|-a^{*}\leq x\leq a^{*},-b^{*}\leq y\leq b^{*}\right\}$.

### 2.2. Statistical Characteristics of Impact Load

The random process of the impact load as the excitation to membrane can be expressed with space and time variables as

(1) P(x,y,t)=Q(x,y)F(t) 

where Q(x,y) is the component impact load varying with space variable expressed as [15,16]

(2) $$Q(x,y)=\left\{ \begin{array}{cc} {\left[\sin\frac{\pi (x+a^{*})}{2a^{*}}\right]}^{C_{1}}{\left[\sin\frac{\pi (y+b^{*})}{2b^{*}}\right]}^{C_{2}} & \left((x,y)\in \Omega_{0}\right)\\ 0 & \left((x,y)\notin \Omega_{0}\right) \end{array}\right.\;$$

where $C_{1}$ and $C_{2}$ are the spatial shape parameters along the x axis and the y axis, respectively.

**Figure 2:**
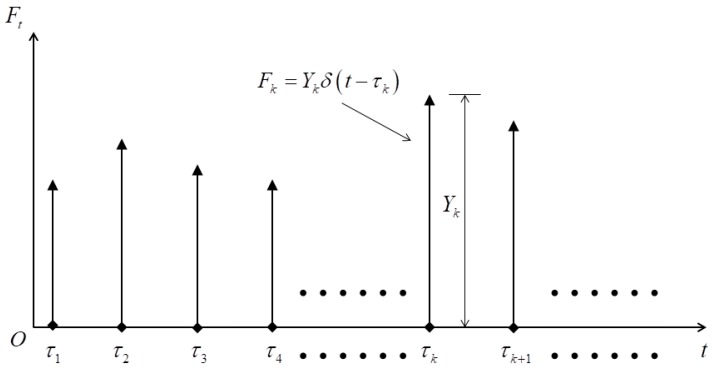
The model of random pulse impact load.

Based on the characteristics of impact load in nature, the random impact load in time domain can be extracted and modeled as Figure 2. As the random impact load is consisted of a series of independent impact process, the component impact load varying with time variable can be expressed as

(3) $$F(t)=\sum_{k=1}^{N(t)}Y_{k}\delta \left(t-\tau_{k}\right)\;$$

where $\tau_{k}$ is the moment when the *k*-th impact load applies on membrane, $Y_{k}$ is the corresponding impact stress, $\delta \left(t,\tau_{k}\right)$ is the Dirac function, and N(t) is the counting process assumed as Poisson process. 

In general, the energy of impact load can be transferred mostly into the system, and the reflecting velocity of impact load will show little to be ignored. Thus, the reflecting velocity of impact load is assumed to be zero in this paper. According to the theorem of momentum, the impact stress can be calculated as

(4) $$Y_{k}=\frac{mv_{0}}{S\tau_{0}}\;$$

where $Y_{k}$ is the k-th impact stress; m is the mass of impact load; $v_{0}$ is the velocity of impact load; S is the impact area on membrane surface; and $\tau_{0}$ is the applied time interval of impact load, and can be selected as 0.002 s [17]. 

Herein, it should be noted that only the velocity of impact load is the random variable, which will directly lead to the uncertainty characterization of amplitude of impact load. Therefore, the statistical characteristics of impact load investigated here are in essence related to the random velocity of impact load. 

According to the definition of Poisson process, the probabilistic function of the counting process N(t) within the time interval [0,T] can be expressed as

(5) $$P\left(n,T\right)=\frac{1}{n!}{\left[\int_{0}^{T}\lambda \left(\tau \right)d\tau \right]}^{n}\exp\left[-\int_{0}^{T}\lambda \left(\tau \right)d\tau \right]\;$$

where λ(τ) is the arrival rate of Poisson process.

Then, the mean function of random impact load can be derived further based on the conditional probabilistic rule.

(6) $$\begin{array}{cc} E\left[p(t)\right]= & E\left[\sum_{k=1}^{N(T)}Y_{k}\delta (t,\tau_{k})\right]\\ & =E\left[E\left[\sum_{k=1}^{N(T)}Y_{k}\delta (t,\tau_{k})|N\left(T\right)\right]\right]\\ & =\sum_{n=0}^{\infty }P\left(n,T\right)E\left[\sum_{k=1}^{n}Y_{k}\delta (t,\tau_{k})\right] \end{array}\;$$

As the variables of $Y_{k}$ and $\tau_{k}$ are independent, Equation (6) can be simplified as

(7) $$E\left[p(t)\right]=\sum_{n=0}^{\infty }P\left(n,T\right)\sum_{k=1}^{n}E\left[Y_{k}\right]E\left[\delta (t,\tau_{k})\right]\;$$

Based on the definition of mean function and Dirac function, the mean function of $\delta (t,\tau_{k})$ can be expressed as

(8) $$E\left[\delta (t,\tau_{k})\right]=\frac{\int_{0}^{T}\delta \left(t-\tau_{k}\right)\lambda \left(\tau_{k}\right)d\tau_{k}}{\int_{0}^{T}\lambda \left(\tau_{k}\right)d\tau_{k}}\;$$

By substituting Equations (5) and (8) into Equation (7), the mean function of amplitude p(t) can be obtained as

(9) $$\begin{array}{cc} E\left[p(t)\right] & =E\left[Y_{k}\right]\sum_{n=0}^{\infty }\frac{1}{n!}{\left[\int_{0}^{T}\lambda \left(\tau \right)d\tau \right]}^{n}\exp\left[-\int_{0}^{T}\lambda \left(\tau \right)d\tau \right]\frac{n\int_{0}^{T}\delta \left(t-\tau_{k}\right)\lambda \left(\tau_{k}\right)d\tau_{k}}{\int_{0}^{T}\lambda \left(\tau_{k}\right)d\tau_{k}}\\ & =E\left[Y_{k}\right]\sum_{n=1}^{\infty }\frac{1}{\left(n-1\right)!}{\left[\int_{0}^{T}\lambda \left(\tau \right)d\tau \right]}^{n-1}\exp\left[-\int_{0}^{T}\lambda \left(\tau \right)d\tau \right]\int_{0}^{T}\delta \left(t-\tau_{k}\right)\lambda \left(\tau_{k}\right)d\tau_{k} \end{array}\;$$

By extending $\exp\left[\int_{0}^{T}\lambda \left(\tau \right)d\tau \right]$ as the Taylor series,

(10) $$\exp\left[\int_{0}^{T}\lambda \left(\tau \right)d\tau \right]=\sum_{n=1}^{\infty }\frac{1}{\left(n-1\right)!}{\left[\int_{0}^{T}\lambda \left(\tau \right)d\tau \right]}^{n-1}\;$$

And substituting Equation (10) into Equation (9), the mean value of amplitude p(t) can be identified as

(11) $$\begin{array}{cc} E\left[p(t)\right] & =E\left[Y_{k}\right]\int_{0}^{T}\delta \left(t-\tau_{k}\right)\lambda \left(\tau_{k}\right)d\tau_{k}\\ & =E\left[Y_{k}\right]\lambda \left(\tau_{k}\right) \end{array}\;$$

The auto-correlated function of amplitude p(t) can be expressed as

(12) $$\begin{array}{cc} \phi_{pp}\left(t_{1},t_{2}\right) & =E\left[p\left(t_{1}\right)p\left(t_{2}\right)\right]\\ & =E\left[\sum_{k=1}^{N(T)}\sum_{l=1}^{N(T)}Y_{k}\delta (t_{1},\tau_{k})Y_{l}\delta (t_{2},\tau_{l})\right]\\ & =\sum_{n=0}^{\infty }P\left(n,T\right)\sum_{k=1}^{N(T)}\sum_{l=1}^{N(T)}E\left[Y_{k}Y_{l}\right]E\left[\delta (t_{1},\tau_{k})\delta (t_{2},\tau_{l})\right]\\ & =\sum_{n=0}^{\infty }P\left(n,T\right)\left\{\begin{array}{l} \sum_{k=1\left(k=l\right)}^{N(T)}E\left[Y_{k}^{2}\right]E\left[\delta (t_{1},\tau_{k})\delta (t_{2},\tau_{l})\right]+\\ \sum_{k=1}^{N(T)}\sum_{l=1}^{N(T)}E\left[Y_{k}Y_{l}\right]E\left[\delta (t_{1},\tau_{k})\delta (t_{2},\tau_{l})\right] \end{array}\right\} \end{array}\;$$

By substituting the Equations (5), (8), and (10) into Equation (12), the auto-correlated function of amplitude can be identified as

(13) $$\phi_{pp}\left(t_{1},t_{2}\right)=E\left[Y_{k}^{2}\right]\lambda \left(t_{1}\right)\delta \left(t_{2}-t_{1}\right)+{\left[E\left(Y_{k}\right)\right]}^{2}\lambda \left(t_{1}\right)\lambda \left(t_{2}\right)\;$$

When $t_{1}=t_{2}=t$ in Equation (13), the mean square function can be obtained as

(14) $$E\left[p^{2}\left(t\right)\right]=E\left[Y_{k}^{2}\right]\lambda \left(t\right)\delta \left(0\right)+{\left[E\left(Y_{k}\right)\right]}^{2}\lambda^{2}\left(t\right)\;$$

Furthermore, the covariance function of amplitude can be derived as

(15) $$\begin{array}{cc} \mathrm{cov}\left(p(t_{1}),p(t_{2})\right)= & E\left[p(t_{1})p(t_{2})\right]-E\left[p(t_{1})\right]E\left[p(t_{2})\right]\\ & =\phi_{pp}\left(t_{1},t_{2}\right)-{\left[E\left(Y_{k}\right)\right]}^{2}\lambda \left(t_{1}\right)\lambda \left(t_{2}\right)\\ & =E\left[Y_{k}^{2}\right]\lambda \left(t_{1}\right)\delta \left(t_{2}-t_{1}\right) \end{array}\;$$

When $t_{1}=t_{2}=t$ in Equation (15), the variance function of amplitude can be obtained as

(16) $$\sigma_{p}^{2}\left(t\right)=E\left[Y_{k}^{2}\right]\lambda \left(t\right)\delta \left(0\right)\;$$

According to the Wiener–Khinchin theorem [18], the power spectral density (PSD) function can be obtained by means of Fourier Transform.

(17) $$\begin{array}{cc} S\left(\omega \right) & =\frac{1}{2\pi }\int_{-\infty }^{\infty }\phi_{pp}\left(t_{1},t_{2}\right)e^{-i\omega \tau }d\tau \\ & =\frac{1}{2\pi }\int_{-\infty }^{\infty }\left\{E\left[Y_{k}^{2}\right]\lambda \left(t_{1}\right)\delta \left(t_{2}-t_{1}\right)+{\left[E\left(Y_{k}\right)\right]}^{2}\lambda \left(t_{1}\right)\lambda \left(t_{2}\right)\right\}e^{-i\omega \tau }d\tau \\ & =\frac{E\left[Y_{k}^{2}\right]\lambda \left(t\right)}{2\pi }+{\left[E\left(Y_{k}\right)\right]}^{2}\lambda^{2}\left(t\right)\delta \left(\omega \right) \end{array}\;$$

As the random impact process induced by impact load is mutually independent, the random impact process can be assumed as a stationary process herein. Consequently, the variables including arrival time λ(τ), mean value of impact stress $E\left(Y_{k}\right)$, and mean square value of impact stress $E\left[Y_{k}^{2}\right]$ are constant, and we note them as

(18) $$\lambda \left(t\right)=\lambda_{0}{,}_{}E\left[Y_{k}\right]=\mu_{Y}{,}_{}E\left[Y_{k}^{2}\right]={\mu^{\prime }}_{Y}\;$$

By substituting Equation (18) into Equation (11), the mean function could be determined as

(19) $$E\left[p(t)\right]=\mu_{Y}\lambda_{0}\;$$

Substituting Equation (18) and noting $\tau =t_{2}-t_{1}$ yields the auto-correlated function,

(20) $$\phi_{pp}\left(\tau \right)={\mu^{\prime }}_{Y}\lambda_{0}\delta \left(\tau \right)+\mu_{Y}^{2}\lambda_{0}^{2}\;$$

When $t_{1}=t_{2}$ in Equation (20), the mean square function can be found as

(21) $$E\left[p^{2}\left(t\right)\right]={\mu^{\prime }}_{Y}\lambda_{0}\delta \left(0\right)+\mu_{Y}^{2}\lambda_{0}^{2}\;$$

By substituting $\tau =t_{2}-t_{1}$ into Equation (15), the covariance function can be determined as

(22) $$\mathrm{cov}\left(p(t_{1}),p(t_{2})\right)={\mu^{\prime }}_{Y}\lambda_{0}\delta \left(\tau \right)\;$$

When $t_{1}=t_{2}$ in Equation (22), the variance function can be found as

(23) $$\sigma_{p}^{2}\left(t\right)={\mu^{\prime }}_{Y}\lambda_{0}\delta \left(0\right)\;$$

By substituting Equation (18) into Equation (17), the power spectral density function can be determined as

(24) $$S\left(\omega \right)=\frac{{\mu^{\prime }}_{Y}\lambda_{0}}{2\pi }+\mu_{Y}^{2}\lambda_{0}^{2}\delta \left(\omega \right)\;$$

Based on the statistical results presented above, we find that the statistical properties of the random impact process do not change over time, only varying the time interval.

**Figure 3:**
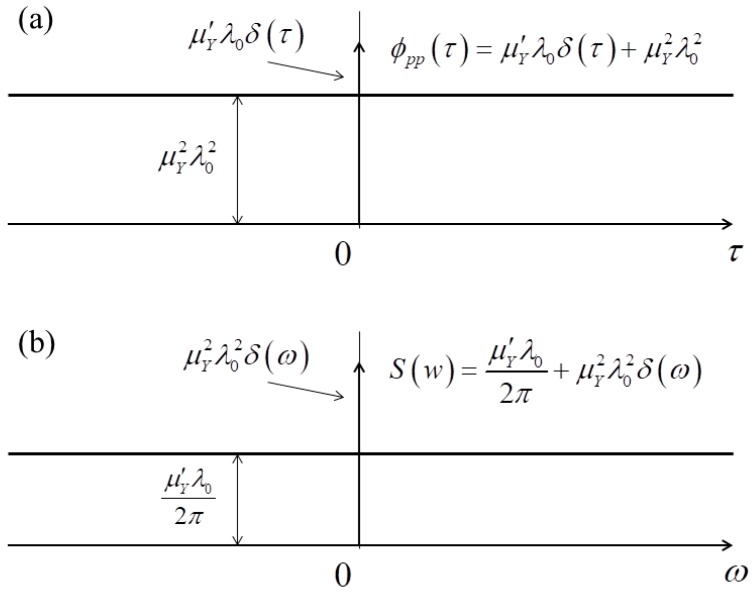
The statistical function of random impact load. (**a**) Auto-correlation function; (**b**) power spectral density function.

After the derivation, the auto-correlation and power density spectrum of the amplitude of random impact load is plotted in Figure 3. It can be found that the spectrum of the impulse process shows a constant, inversely, the spectrum of the constant shows an impulse. For the random impact load model, the power density spectrum of the amplitude, assumed as Poisson process, is uniform except at ω=0, which indicates the random impact load could be modeled as the white noise signal input into the membrane system. For practical engineering, the bandwidth of white noise signal varies within limited interval. Combined with the assumption of amplitude following the Gaussian distribution, the continuous impulse process induced by random impact load can be modeled as white Gaussian noise input into membrane system.

### 2.3. Establishing the FPK Equation

As large deformation may cause the median surface to stretch, the Von Karman theory [19] should be applied to introduce the geometrical equation, to second-order, expressed as

(25) $$\left\{ \begin{array}{l} \varepsilon_{x}=\frac{\partial u}{\partial x}+\frac{1}{2}{\left(\frac{\partial w}{\partial x}\right)}^{2}\\ \varepsilon_{y}=\frac{\partial v}{\partial y}+\frac{1}{2}{\left(\frac{\partial w}{\partial y}\right)}^{2}\\ \gamma_{xy}=\frac{\partial v}{\partial x}+\frac{\partial u}{\partial y}+\frac{\partial w}{\partial x}\frac{\partial w}{\partial y} \end{array}\right.\;$$

where $\varepsilon_{x}$ and $\varepsilon_{y}$ are strains in x and y direction, respectively; $\gamma_{xy}$ is shear strain; u and v are displacement in x and y direction, respectively; and w is displacement of out-plane vibration on membrane.

The relation between internal force and stress can be expressed as

(26) $$\left\{ \begin{array}{l} N_{x}=\sigma_{x}h\\ N_{y}=\sigma_{y}h\\ N_{xy}=\tau_{xy}h \end{array}\right.\;$$

where $N_{x}$ and $N_{y}$ are internal stretching force in x and y direction, respectively; $N_{xy}$ is internal shear force; $\sigma_{x}$ and $\sigma_{y}$ are tensile stress in x and y direction, respectively; $\tau_{xy}$ is shear stress; and h is thickness of membrane.

With consideration of orthotropic aspect of membrane material, the physical equation can be expressed as

(27) $$\left\{ \begin{array}{l} \varepsilon_{x}=\frac{1}{E_{1}}(\sigma_{x}-\upsilon_{1}\sigma_{y})=\frac{1}{E_{1}h}(N_{x}-\upsilon_{1}N_{y})\\ \varepsilon_{y}=\frac{1}{E_{2}}(\sigma_{y}-\upsilon_{2}\sigma_{x})=\frac{1}{E_{2}h}(N_{y}-\upsilon_{2}N_{x})\\ \gamma_{xy}=\frac{\tau_{xy}}{G} \end{array}\right.\;$$

where $E_{1}$ and $E_{2}$ are elastic modulus in x and y direction, respectively; G denotes the shear modulus; and $\upsilon_{1}$ and $\upsilon_{2}$ are Poisson’s ratios in x and y direction, respectively. Moreover, the relation between elastic modulus and Poisson’s ratio can be expressed as

(28) $$\frac{\upsilon_{1}}{E_{1}}=\frac{\upsilon_{2}}{E_{2}}\;$$

As the membrane is a perfectly flexible thin plate, it is generally assumed that it provides no resistance to bending moments and the ensuing normal shear forces [20]. Based on the mechanical theory shown above, the governing equations for the vibration of pre-stressed rectangular membrane have been derived by Zheng et al. [12] and Eisley [21], expressed as

(29) $$\left\{ \begin{array}{l} \rho \frac{\partial^{2}w}{\partial t^{2}}+c\frac{\partial w}{\partial t}-(N_{x}+N_{0x})\frac{\partial^{2}w}{\partial x^{2}}-(N_{y}+N_{0y})\frac{\partial^{2}w}{\partial y^{2}}=P(x,y,t)\\ \frac{1}{E_{1}h}\frac{\partial^{2}N_{x}}{\partial y^{2}}+\frac{1}{E_{2}h}\frac{\partial^{2}N_{y}}{\partial x^{2}}={\left(\frac{\partial^{2}w}{\partial x\partial y}\right)}^{2}-\frac{\partial^{2}w}{\partial x^{2}}{\frac{\partial^{2}w}{\partial y^{2}}}_{}^{}{{}_{}^{}}_{}^{}{{}_{}^{}}_{}^{}{{}_{}^{}}_{}^{}{{}_{}^{}}_{}^{}{{}_{}^{}}_{}^{}{{}_{}^{}}_{}^{}{{}_{}^{}}_{}^{}{{}_{}^{}}_{}^{} \end{array}\right.\;$$

where c is coefficient of damping.

The boundary condition of displacement can be expressed as

(30) $$\left\{ \begin{array}{l} w\left(x,b,t\right)=0,\;w\left(a,y,t\right)=0\\ w\left(x,-b,t\right)=0,\;w\left(-a,y,t\right)=0\; \end{array}\right.\;\;$$

By introducing the Airy stress function φ(x,y,t), the relation between the membrane force and the Airy stress function can be expressed as

(31) $$\left\{ \begin{array}{l} N_{x}=h\frac{\partial^{2}\varphi }{\partial y^{2}}\\ N_{y}=h\frac{\partial^{2}\varphi }{\partial x^{2}} \end{array}\right.\;$$

In view of the boundary conditions, the mode shape function can be selected as

(32) $$W(x,y)=\sum_{m=1}^{\infty }\sum_{n=1}^{\infty }\sin\frac{m\pi (x+a)}{2a}\sin\frac{n\pi (y+b)}{2b}\;$$

where m and n are the numbers of half-waves along the x and y direction, respectively.

The displacement function which satisfies Equation (30) can be assumed as

(33) $$\begin{array}{cc} w(x,y,t) & =\sum_{m=1}^{\infty }\sum_{n=1}^{\infty }T_{mn}(t)W_{mn}(x,y)\\ & =\sum_{m=1}^{\infty }\sum_{n=1}^{\infty }T_{mn}(t)\sin\frac{m\pi \left(x+a\right)}{2a}\sin\frac{n\pi \left(y+b\right)}{2b} \end{array}\;$$

The Airy stress function can be separated [12] as

(34) $$\varphi (x,y,t)=\sum_{m=1}^{\infty }\sum_{n=1}^{\infty }T^{2}{}_{mn}(t)\phi_{mn}(x,y)\;$$

where T(t) is time function and ϕ(x,y) is space function.

After the substitution of Equation (34) into Equation (29), the following formula can be obtained as

(35) $$\frac{1}{E_{1}}\frac{\partial^{4}\phi }{\partial y^{4}}+\frac{1}{E_{2}}\frac{\partial^{4}\phi }{\partial x^{4}}=\frac{m^{2}n^{2}\pi^{4}}{32a^{2}b^{2}}\left(\cos\frac{m\pi (x+a)}{a}+\cos\frac{n\pi (y+b)}{b}\right)\;$$

In the view of the boundary condition, the solution of Equation (35) can be assumed as

(36) $$\phi (x,y)=\alpha \cos\frac{m\pi (x+a)}{a}+\beta \cos\frac{n\pi (x+a)}{a}+\gamma_{1}x^{3}+\gamma_{2}x^{2}+\gamma_{3}y^{3}+\gamma_{4}y^{2}\;$$

By substituting Equation (36) into Equation (35), the undetermined parameters can be obtained as

(37) $$\alpha ={\frac{E_{2}n^{2}a^{2}}{32m^{2}b^{2}}}_{}^{}{}_{}^{},\beta ={\frac{E_{1}m^{2}b^{2}}{32n^{2}a^{2}}}_{}^{}{}_{}^{},\gamma_{1}=\gamma_{3}=0{,}_{}{}_{}\gamma_{2}=\frac{\pi^{2}n^{2}E_{2}}{64b^{2}}{,}_{}{}_{}\gamma_{4}=\frac{\pi^{2}m^{2}E_{1}}{64a^{2}}\;$$

By substituting Equations (34), (36), and (37) into Equation (29), the governing Equation (29) can be decoupled by the Galerkin method [22] and expressed as

(38) $$\begin{array}{l} \int_{-a}^{a}\int_{-b}^{b}\left[\begin{array}{l} \rho W^{2}\frac{d^{2}T(t)}{dt^{2}}+cW^{2}\frac{dT(t)}{dt}-W\left(N_{0x}\frac{\partial^{2}W}{\partial x^{2}}+N_{0y}\frac{\partial^{2}W}{\partial y^{2}}\right)T(t)\\ -hW\left(\frac{\partial^{2}\phi }{\partial y^{2}}\frac{\partial^{2}W}{\partial x^{2}}+\frac{\partial^{2}\phi }{\partial x^{2}}\frac{\partial^{2}W}{\partial y^{2}}\right)T^{3}(t) \end{array}\right]dxdy\\ =\int_{-a}^{a}\int_{-b}^{b}\left[WQ(x,y)F(t)\right]dxdy \end{array}\;$$

After integration of Equation (38), the inhomogeneous algebraic equation can be obtained as

(39) $$\frac{d^{2}T(t)}{dt^{2}}+\frac{c}{\rho }\frac{dT(t)}{dt}+\frac{\pi^{2}b^{2}N_{0x}+\pi^{2}a^{2}N_{0y}}{4\rho a^{2}b^{2}}T(t)+\frac{3\pi^{4}h\beta +3\pi^{4}h\alpha }{8\rho a^{2}b^{2}}T^{3}(t)=Kp(t)\;$$

where

$$K=\int_{-a}^{a}\int_{-b}^{b}\left\{{\left[\sin\frac{\pi (x+a^{*})}{2a^{*}}\right]}^{C_{1}}{\left[\sin\frac{\pi (y+b^{*})}{2b^{*}}\right]}^{C_{2}}\sin\frac{m\pi \left(x+a\right)}{2a}\sin\frac{n\pi \left(y+b\right)}{2b}\right\}dxdy\;$$

Then, Equation (39) can be simplified as

(40) $$\ddot{X}+2n_{0}\dot{X}+w_{0}^{2}X+\mu_{0}X^{3}=Kp(t)\;$$

where $\ddot{X}=\frac{d^{2}T(t)}{dt^{2}}{,}_{}\dot{X}=\frac{dT(t)}{dt}{,}_{}2n_{0}=\frac{c}{\rho }{,}_{}w_{0}^{2}=\frac{\pi^{2}b^{2}N_{0x}+\pi^{2}a^{2}N_{0y}}{4\rho a^{2}b^{2}}{,}_{}\mu_{0}=\frac{3\pi^{4}h(\alpha +\beta )}{8\rho a^{2}b^{2}}$.

It can be found from Equation (40) that the governing motion equation [Equation (39)] has been decoupled by the Galerkin technique, and transferred into the Duffing equation. For this simplified random vibration model, the FPK method could be applied to obtain the exact analytical solution. As Equation (40) is intended to be solved by the FPK method, the expression of Equation (40) should firstly be turned into the expression of Ito equation. Note that

(41) $$\left\{ \begin{array}{l} \dot{X}=Y_{1}\\ X=Y_{2} \end{array}\right.\;$$

Then, Equation (40) can be transformed into Ito equation as

(42) $$\left\{ \begin{array}{l} {\dot{Y}}_{1}=-2n_{0}Y_{1}-w_{0}^{2}Y_{2}-\mu_{0}Y_{2}^{3}+Kp(t)\\ {\dot{Y}}_{2}=Y_{1} \end{array}\right.\;$$

Based on the random impact load model proposed above, the statistical results of load term Kp(t) in Equation (40) can be obtained as

(43) $$\mathrm{mean}\;\mathrm{function}:\;E\left[Kp(t)\right]=\mu_{Y}\lambda_{0}K\;$$

Further, the auto-correlated function is

(44) $$E\left[Kp(t)Kp(t+\tau )\right]=K^{2}\left[{\mu^{\prime }}_{Y}\lambda_{0}\delta \left(\tau \right)+\mu_{Y}^{2}\lambda_{0}^{2}\right]\;$$

Because Equation (42) is Ito equation, whose solutions possess the Markovian characteristics, the stationary condition is ∂p/∂t=0 [23]. Then, the FPK equation derived by Er [24] can be applied to establish the FPK equation of stochastic vibration for membrane as

(45) $$\frac{\partial (a_{1}p)}{\partial Y_{1}}+\frac{\partial (a_{2}p)}{\partial Y_{2}}-\frac{1}{2}\frac{\partial^{2}(b_{11}p)}{\partial Y_{1}^{2}}-\frac{1}{2}\frac{\partial^{2}(b_{12}p)}{\partial Y_{1}\partial Y_{2}}-\frac{1}{2}\frac{\partial^{2}(b_{21}p)}{\partial Y_{2}\partial Y_{1}}-\frac{1}{2}\frac{\partial^{2}(b_{22}p)}{\partial Y_{2}^{2}}=0\;$$

where $p=p(Y_{1},Y_{2})$ is the joint probability density function of variables $Y_{1}$ and $Y_{2}$.

By substituting Equation (43) into Equation (42), the drift coefficient $a_{1}$ in Equation (45) can be obtained as

(46) $$a_{1}=\;\underset{\Delta t\to 0}{\lim}\frac{1}{\Delta t}E\left[Y_{1}(t+\Delta t)-Y_{1}(t)\right]=-2n_{0}Y_{1}-w_{0}^{2}Y_{2}-\mu_{0}Y_{2}^{3}+\mu_{Y}\lambda_{0}K\;$$

Based on Equation (42), we can derive the drift coefficients $a_{2}$ in Equation (45) as

(47) $$a_{2}=\underset{\Delta t\to 0}{\lim}\frac{1}{\Delta t}E\left[Y_{2}(t+\Delta t)-Y_{2}(t)\right]=Y_{1}\;$$

Then, by substituting Equation (44) into Equation (42), the diffusion coefficients $b_{11}$ in Equation (45) can be obtained as

(48) $$b_{11}=\underset{\Delta t\to 0}{\lim}\frac{1}{\Delta t}E\left\{{\left[Y_{1}(t+\Delta t)-Y_{1}(t)\right]}^{2}\right\}={\mu^{\prime }}_{Y}\lambda_{0}K^{2}\;$$

Substituting Equation (44) into (42) can yield the diffusion coefficients $b_{21}$ and $b_{12}$ in Equation (45), expressed as

(49) $$b_{12}=b_{21}=\underset{\Delta t\to 0}{\lim}\frac{1}{\Delta t}E\left[\left[Y_{1}(t+\Delta t)-Y_{1}(t)\right]\left[Y_{2}(t+\Delta t)-Y_{2}(t)\right]\right]=0\;$$

By substituting Equation (44) into (42), the diffusion coefficient diffusion coefficients $b_{22}$ in Equation (45) can be expressed as

(50) $$b_{22}=\underset{\Delta t\to 0}{\lim}\frac{1}{\Delta t}E\left\{{\left[Y_{2}(t+\Delta t)-Y_{2}(t)\right]}^{2}\right\}=0\;$$

By substituting Equations (46)–(50) into Equation (45), the FPK equation (45) can be transformed into

(51) $$-n_{0}Y_{1}\frac{\partial p}{\partial Y_{1}}-\left(w_{0}^{2}Y_{2}+\mu_{0}Y_{2}^{3}-\mu_{Y}\lambda_{0}K\right)\frac{\partial p}{\partial Y_{1}}+Y_{1}\frac{\partial p}{\partial Y_{2}}-\frac{{\mu^{\prime }}_{Y}\lambda_{0}K^{2}}{2}\frac{\partial^{2}p}{\partial Y_{1}^{2}}=0\;$$

In order to solve the FPK equation (45), Equation (51) can be collected as

(52) $$\left[Y_{1}\frac{\partial p}{\partial Y_{2}}-\left(w_{0}^{2}Y_{2}+\mu_{0}Y_{2}^{3}-\mu_{Y}\lambda_{0}K\right)\frac{\partial p}{\partial Y_{1}}\right]-\frac{\partial }{\partial Y_{1}}\left(n_{0}Y_{1}p+\frac{{\mu^{\prime }}_{Y}\lambda_{0}K^{2}}{2}\frac{\partial p}{\partial Y_{1}}\right)=0\;$$

By neglecting the second term in Equation (52), we can obtain the analytical solution approximately as

(53) $$Y_{1}\frac{\partial p}{\partial Y_{2}}-\left(w_{0}^{2}Y_{2}+\mu_{0}Y_{2}^{3}-\mu_{Y}\lambda_{0}K\right)\frac{\partial p}{\partial Y_{1}}=0\;$$

Moreover, the approximation herein is expected to be verified by the further experimental study.

### 2.4. Solving the FPK Equation

For stationary process, the response variable Y and its derivative variable $\dot{Y}$ are independent [25,26]. Then, the variables $Y_{1}$ and $Y_{2}$ in Equation (45) are independent as well, and the joint probability density function can be decoupled as

(54) $$p(Y_{1},Y_{2})=p(Y_{1})p(Y_{2})\;$$

Then, the probably density function can be turned into the one-dimensional variable. Thus, Equation (53) can be transformed into

(55) $$-n_{0}-\left(w_{0}^{2}Y_{2}+\mu_{0}Y_{2}^{3}-\mu_{Y}\lambda_{0}K\right)\frac{{p^{\prime }}_{1}\left(Y_{1}\right)}{p_{1}\left(Y_{1}\right)}+Y_{1}\frac{{p^{\prime }}_{2}\left(Y_{2}\right)}{p_{2}\left(Y_{2}\right)}-\frac{{\mu^{\prime }}_{Y}\lambda_{0}K^{2}}{2}\frac{{p^{\prime \prime }}_{1}\left(Y_{1}\right)}{p_{1}\left(Y_{1}\right)}=0\;$$

Further, Equation (53) can be transformed into

(56) $$\left\{ \begin{array}{l} \frac{1}{Y_{1}}\frac{{p^{\prime }}_{1}\left(Y_{1}\right)}{p_{1}\left(Y_{1}\right)}=-L\\ \frac{1}{\left(w_{0}^{2}Y_{2}+\mu_{0}Y_{2}^{3}-\mu_{Y}\lambda_{0}K\right)}\frac{{p^{\prime }}_{2}\left(Y_{2}\right)}{p_{2}\left(Y_{2}\right)}=-L \end{array}\right.\;$$

where L is the undetermined constant.

After integration of Equation (56), the probabilistic density function of variables $Y_{1}$ and $Y_{2}$ can be obtained, respectively, as

(57) $$\left\{ \begin{array}{l} p_{1}\left(Y_{1}\right)=C_{1}\exp\left(-\frac{LY_{1}^{2}}{2}\right)\\ p_{2}\left(Y_{2}\right)=C_{2}\exp\left[-L\left(\frac{w_{0}^{2}Y_{2}^{2}}{2}+\frac{\mu_{0}Y_{2}^{4}}{4}-\mu_{Y}\lambda_{0}KY_{2}\right)\right] \end{array}\right.\;$$

where $C_{1}$ and $C_{2}$ are the undetermined constants.

By substituting Equation (57) into Equation (55), the undetermined constant L in Equation (56) can be identified as

(58) $$L=\frac{2n_{0}}{{\mu^{\prime }}_{Y}\lambda_{0}K^{2}}\;$$

According to the definition of probabilistic function of $p_{1}\left(Y_{1}\right)$ expressed as

(59) $$\int_{-\infty }^{\infty }p_{1}\left(Y_{1}\right)dY_{1}=1\;$$

Then, the undetermined coefficient $C_{1}$ in Equation (57) can be identified as

(60) $$C_{1}={\left[\frac{{\mu^{\prime }}_{Y}\lambda_{0}K^{2}}{4\pi n_{0}}\right]}^{1/2}\;$$

Finally, the probabilistic density function of $p_{1}\left(Y_{1}\right)$ in Equation (57), namely the velocity variable of dynamic response, can be obtained as

(61) $$p_{1}\left(Y_{1}\right)={\left[\frac{{\mu^{\prime }}_{Y}\lambda_{0}K^{2}}{4\pi n_{0}}\right]}^{1/2}\exp\left(-\frac{n_{0}}{{\mu^{\prime }}_{Y}\lambda_{0}K^{2}}Y_{1}^{2}\right)\;$$

Consequently, the mean and variance of velocity can be obtained, respectively, as

(62) $$\mu_{Y_{1}}=\int_{-\infty }^{\infty }p_{1}\left(Y_{1}\right)Y_{1}dY_{1}=0\;$$

and

(63) $$\begin{array}{cc} \sigma_{Y_{1}}^{2} & =\int_{-\infty }^{\infty }p_{1}\left(Y_{1}\right)Y_{1}^{2}dY_{1}\\ & =\frac{{\mu^{\prime }}_{Y}\lambda_{0}K^{2}}{2n_{0}} \end{array}\;$$

Extend $\exp\left(-\mu_{0}LY_{2}^{4}/4\right)$ in Equation (57) as the Taylor series, and select the first two terms as

(64) $$\exp\left(-\frac{\mu_{0}LY_{2}^{4}}{4}\right)=1-\frac{\mu_{0}LY_{2}^{4}}{4}+o\left(Y_{2}\right)\;$$

By substituting Equation (64) into Equation (57), and introducing the definition of probabilistic function of $p_{2}\left(Y_{2}\right)$, expressed as

(65) $$\int_{-\infty }^{\infty }p_{2}\left(Y_{2}\right)dY_{2}=1\;$$

Then, the undetermined coefficient $C_{2}$ in Equation (57) can be identified as

(66) $$C_{2}={\left\{\exp\left(\frac{n_{0}\mu_{Y}^{2}}{{\mu^{\prime }}_{Y}w_{0}^{2}}\right)\left[\sqrt{\frac{\pi {\mu^{\prime }}_{Y}\lambda_{0}K^{2}}{n_{0}w_{0}^{2}}}-\frac{\mu_{0}n_{0}}{2{\mu^{\prime }}_{Y}\lambda_{0}K^{2}}\left(\begin{array}{l} {\left(\frac{\mu_{Y}^{}\lambda_{0}^{}K}{w_{0}^{2}}\right)}^{4}\sqrt{\frac{\pi {\mu^{\prime }}_{Y}\lambda_{0}K^{2}}{n_{0}w_{0}^{2}}}\\ +3\sqrt{\pi }{\left(\frac{\mu_{Y}^{}\lambda_{0}^{}K}{w_{0}^{2}}\right)}^{2}{\left(\frac{n_{0}w_{0}^{2}}{{\mu^{\prime }}_{Y}\lambda_{0}K^{2}}\right)}^{-3/2}\\ +\frac{3\sqrt{\pi }}{4}{\left(\frac{n_{0}w_{0}^{2}}{{\mu^{\prime }}_{Y}\lambda_{0}K^{2}}\right)}^{-5/2} \end{array}\right)\right]\right\}}^{-1}\;$$

Finally, the probabilistic density function of $p_{2}\left(Y_{2}\right)$ in Equation (57), namely the displacement variable of dynamic response at Point O, can be obtained as

(67) $$\begin{array}{cc} p_{2}\left(Y_{2}\right)= & {\left\{\exp\left(\frac{n_{0}\mu_{Y}^{2}}{{\mu^{\prime }}_{Y}w_{0}^{2}}\right)\left[\sqrt{\frac{\pi {\mu^{\prime }}_{Y}\lambda_{0}K^{2}}{n_{0}w_{0}^{2}}}-\frac{\mu_{0}n_{0}}{2{\mu^{\prime }}_{Y}\lambda_{0}K^{2}}\left(\begin{array}{l} {\left(\frac{\mu_{Y}^{}\lambda_{0}^{}K}{w_{0}^{2}}\right)}^{4}\sqrt{\frac{\pi {\mu^{\prime }}_{Y}\lambda_{0}K^{2}}{n_{0}w_{0}^{2}}}\\ +3\sqrt{\pi }{\left(\frac{\mu_{Y}^{}\lambda_{0}^{}K}{w_{0}^{2}}\right)}^{2}{\left(\frac{n_{0}w_{0}^{2}}{{\mu^{\prime }}_{Y}\lambda_{0}K^{2}}\right)}^{-3/2}\\ +\frac{3\sqrt{\pi }}{4}{\left(\frac{n_{0}w_{0}^{2}}{{\mu^{\prime }}_{Y}\lambda_{0}K^{2}}\right)}^{-5/2} \end{array}\right)\right]\right\}}^{-1}\\ & \cdot \left(1-\frac{\mu_{0}n_{0}Y_{2}^{4}}{2{\mu^{\prime }}_{Y}\lambda_{0}K^{2}}\right)\exp\left[-\frac{n_{0}w_{0}^{2}Y_{2}^{2}}{{\mu^{\prime }}_{Y}\lambda_{0}K^{2}}+\frac{2n_{0}\mu_{Y}Y_{2}}{{\mu^{\prime }}_{Y}K}\right] \end{array}\;$$

By substituting Equations (67) into Equation (33), the probabilistic density function of displacement at any point on membrane surface can be determined as

(68) $$\begin{array}{cc} p_{2}\left(Y_{2}\right)= & \sum_{m=1}^{\infty }\sum_{n=1}^{\infty }\sin\frac{m\pi x}{a}\sin\frac{n\pi y}{b}{\left\{\exp\left(\frac{n_{0}\mu_{Y}^{2}}{{\mu^{\prime }}_{Y}w_{0}^{2}}\right)\left[\sqrt{\frac{\pi {\mu^{\prime }}_{Y}\lambda_{0}K^{2}}{n_{0}w_{0}^{2}}}-\frac{\mu_{0}n_{0}}{2{\mu^{\prime }}_{Y}\lambda_{0}K^{2}}\left(\begin{array}{l} {\left(\frac{\mu_{Y}^{}\lambda_{0}^{}K}{w_{0}^{2}}\right)}^{4}\sqrt{\frac{\pi {\mu^{\prime }}_{Y}\lambda_{0}K^{2}}{n_{0}w_{0}^{2}}}\\ +3\sqrt{\pi }{\left(\frac{\mu_{Y}^{}\lambda_{0}^{}K}{w_{0}^{2}}\right)}^{2}{\left(\frac{n_{0}w_{0}^{2}}{{\mu^{\prime }}_{Y}\lambda_{0}K^{2}}\right)}^{-3/2}\\ +\frac{3\sqrt{\pi }}{4}{\left(\frac{n_{0}w_{0}^{2}}{{\mu^{\prime }}_{Y}\lambda_{0}K^{2}}\right)}^{-5/2} \end{array}\right)\right]\right\}}^{-1}\\ & \cdot \left(1-\frac{\mu_{0}n_{0}Y_{2}^{4}}{2{\mu^{\prime }}_{Y}\lambda_{0}K^{2}}\right)\exp\left[-\frac{n_{0}w_{0}^{2}Y_{2}^{2}}{{\mu^{\prime }}_{Y}\lambda_{0}K^{2}}+\frac{2n_{0}\mu_{Y}Y_{2}}{{\mu^{\prime }}_{Y}K}\right] \end{array}\;$$

Consequently, the mean and variance of displacement can be obtained respectively as

(69) $$\begin{array}{l} \mu_{Y_{2}}=\int_{-\infty }^{\infty }p_{2}\left(Y_{2}\right)Y_{2}dY_{2}\\ ={\left[\sqrt{\frac{\pi {\mu^{\prime }}_{Y}\lambda_{0}K^{2}}{n_{0}w_{0}^{2}}}-\frac{\mu_{0}n_{0}}{2{\mu^{\prime }}_{Y}\lambda_{0}K^{2}}\left(\begin{array}{l} {\left(\frac{\mu_{Y}^{}\lambda_{0}^{}K}{w_{0}^{2}}\right)}^{4}\sqrt{\frac{\pi {\mu^{\prime }}_{Y}\lambda_{0}K^{2}}{n_{0}w_{0}^{2}}}\\ +3\sqrt{\pi }{\left(\frac{\mu_{Y}^{}\lambda_{0}^{}K}{w_{0}^{2}}\right)}^{2}{\left(\frac{n_{0}w_{0}^{2}}{{\mu^{\prime }}_{Y}\lambda_{0}K^{2}}\right)}^{-3/2}+\frac{3\sqrt{\pi }}{4}{\left(\frac{n_{0}w_{0}^{2}}{{\mu^{\prime }}_{Y}\lambda_{0}K^{2}}\right)}^{-5/2} \end{array}\right)\right]}^{-1}\\ {}_{}\cdot \left(\begin{array}{l} -\frac{\mu_{0}n_{0}}{2{\mu^{\prime }}_{Y}\lambda_{0}K^{2}}\left(\begin{array}{l} 15\sqrt{\pi }\frac{\mu_{Y}\lambda_{0}K}{w_{0}^{2}}{\left(\frac{2n_{0}w_{0}^{2}}{{\mu^{\prime }}_{Y}\lambda_{0}K^{2}}\right)}^{-5/2}+10\sqrt{2\pi }{\left(\frac{\mu_{Y}\lambda_{0}K}{w_{0}^{2}}\right)}^{3}{\left(\frac{2n_{0}w_{0}^{2}}{{\mu^{\prime }}_{Y}\lambda_{0}K^{2}}\right)}^{-3/2}\\ +\sqrt{2\pi }{\left(\frac{\mu_{Y}\lambda_{0}K}{w_{0}^{2}}\right)}^{5}{\left(\frac{2n_{0}w_{0}^{2}}{{\mu^{\prime }}_{Y}\lambda_{0}K^{2}}\right)}^{-1/2} \end{array}\right)\\ +\sqrt{2\pi }\frac{\mu_{Y}\lambda_{0}K}{w_{0}^{2}}{\left(\frac{2n_{0}w_{0}^{2}}{{\mu^{\prime }}_{Y}\lambda_{0}K^{2}}\right)}^{-1/2} \end{array}\right) \end{array}\;$$

and

(70) $$\begin{array}{cc} \sigma_{Y_{2}}^{2} & =\int_{-\infty }^{\infty }p_{2}\left(Y_{2}\right)Y_{2}^{2}dY_{2}\\ & ={\left[\sqrt{\frac{\pi {\mu^{\prime }}_{Y}\lambda_{0}K^{2}}{n_{0}w_{0}^{2}}}-\frac{\mu_{0}n_{0}}{2{\mu^{\prime }}_{Y}\lambda_{0}K^{2}}\left(\begin{array}{l} {\left(\frac{\mu_{Y}^{}\lambda_{0}^{}K}{w_{0}^{2}}\right)}^{4}\sqrt{\frac{\pi {\mu^{\prime }}_{Y}\lambda_{0}K^{2}}{n_{0}w_{0}^{2}}}\\ +3\sqrt{\pi }{\left(\frac{\mu_{Y}^{}\lambda_{0}^{}K}{w_{0}^{2}}\right)}^{2}{\left(\frac{n_{0}w_{0}^{2}}{{\mu^{\prime }}_{Y}\lambda_{0}K^{2}}\right)}^{-3/2}+\frac{3\sqrt{\pi }}{4}{\left(\frac{n_{0}w_{0}^{2}}{{\mu^{\prime }}_{Y}\lambda_{0}K^{2}}\right)}^{-5/2} \end{array}\right)\right]}^{-1}\\ & \cdot \left(\begin{array}{l} -\frac{15\mu_{0}n_{0}}{16{\mu^{\prime }}_{Y}\lambda_{0}K^{2}}\frac{\sqrt{\pi }}{{\left(\frac{n_{0}w_{0}^{2}}{{\mu^{\prime }}_{Y}\lambda_{0}K^{2}}\right)}^{7/2}}+\frac{45}{4}{\left(\frac{\mu_{Y}\lambda_{0}K}{w_{0}^{2}}\right)}^{2}\frac{\sqrt{\pi }}{{\left(\frac{n_{0}w_{0}^{2}}{{\mu^{\prime }}_{Y}\lambda_{0}K^{2}}\right)}^{5/2}}\\ +\left(\frac{15}{2}{\left(\frac{\mu_{Y}\lambda_{0}K}{w_{0}^{2}}\right)}^{4}-\frac{1}{2}\right)\frac{\sqrt{\pi }}{{\left(\frac{n_{0}w_{0}^{2}}{{\mu^{\prime }}_{Y}\lambda_{0}K^{2}}\right)}^{3/2}}+\left({\left(\frac{\mu_{Y}\lambda_{0}K}{w_{0}^{2}}\right)}^{2}-\frac{\mu_{0}n_{0}}{2{\mu^{\prime }}_{Y}\lambda_{0}K^{2}}{\left(\frac{\mu_{Y}\lambda_{0}K}{w_{0}^{2}}\right)}^{6}\right)\frac{\sqrt{\pi }}{{\left(\frac{n_{0}w_{0}^{2}}{{\mu^{\prime }}_{Y}\lambda_{0}K^{2}}\right)}^{1/2}} \end{array}\right) \end{array}\;$$

By substituting Equations (69) and (70) into Equation (33), the mean and variance of displacement on membrane surface can be determined as

(71) $$\begin{array}{l} \mu_{w}=\sum_{m=1}^{\infty }\sum_{n=1}^{\infty }\sin\frac{m\pi x}{a}\sin\frac{n\pi y}{b}{\left[\sqrt{\frac{\pi {\mu^{\prime }}_{Y}\lambda_{0}K^{2}}{n_{0}w_{0}^{2}}}-\frac{\mu_{0}n_{0}}{2{\mu^{\prime }}_{Y}\lambda_{0}K^{2}}\left(\begin{array}{l} {\left(\frac{\mu_{Y}^{}\lambda_{0}^{}K}{w_{0}^{2}}\right)}^{4}\sqrt{\frac{\pi {\mu^{\prime }}_{Y}\lambda_{0}K^{2}}{n_{0}w_{0}^{2}}}\\ +3\sqrt{\pi }{\left(\frac{\mu_{Y}^{}\lambda_{0}^{}K}{w_{0}^{2}}\right)}^{2}{\left(\frac{n_{0}w_{0}^{2}}{{\mu^{\prime }}_{Y}\lambda_{0}K^{2}}\right)}^{-3/2}\\ +\frac{3\sqrt{\pi }}{4}{\left(\frac{n_{0}w_{0}^{2}}{{\mu^{\prime }}_{Y}\lambda_{0}K^{2}}\right)}^{-5/2} \end{array}\right)\right]}^{-1}\\ {}_{}\cdot \left(\begin{array}{l} -\frac{\mu_{0}n_{0}}{2{\mu^{\prime }}_{Y}\lambda_{0}K^{2}}\left(\begin{array}{l} 15\sqrt{\pi }\frac{\mu_{Y}\lambda_{0}K}{w_{0}^{2}}{\left(\frac{2n_{0}w_{0}^{2}}{{\mu^{\prime }}_{Y}\lambda_{0}K^{2}}\right)}^{-5/2}+10\sqrt{2\pi }{\left(\frac{\mu_{Y}\lambda_{0}K}{w_{0}^{2}}\right)}^{3}{\left(\frac{2n_{0}w_{0}^{2}}{{\mu^{\prime }}_{Y}\lambda_{0}K^{2}}\right)}^{-3/2}\\ +\sqrt{2\pi }{\left(\frac{\mu_{Y}\lambda_{0}K}{w_{0}^{2}}\right)}^{5}{\left(\frac{2n_{0}w_{0}^{2}}{{\mu^{\prime }}_{Y}\lambda_{0}K^{2}}\right)}^{-1/2} \end{array}\right)\\ +\sqrt{2\pi }\frac{\mu_{Y}\lambda_{0}K}{w_{0}^{2}}{\left(\frac{2n_{0}w_{0}^{2}}{{\mu^{\prime }}_{Y}\lambda_{0}K^{2}}\right)}^{-1/2} \end{array}\right) \end{array}\;$$

and

(72) $$\begin{array}{cc} \sigma_{w}^{2} & ={\left(\sum_{m=1}^{\infty }\sum_{n=1}^{\infty }\sin\frac{m\pi x}{a}\sin\frac{n\pi y}{b}\right)}^{2}{\left[\begin{array}{l} \sqrt{\frac{\pi {\mu^{\prime }}_{Y}\lambda_{0}K^{2}}{n_{0}w_{0}^{2}}}-\frac{\mu_{0}n_{0}}{2{\mu^{\prime }}_{Y}\lambda_{0}K^{2}}\\ \cdot \left(\begin{array}{l} {\left(\frac{\mu_{Y}^{}\lambda_{0}^{}K}{w_{0}^{2}}\right)}^{4}\sqrt{\frac{\pi {\mu^{\prime }}_{Y}\lambda_{0}K^{2}}{n_{0}w_{0}^{2}}}\\ +3\sqrt{\pi }{\left(\frac{\mu_{Y}^{}\lambda_{0}^{}K}{w_{0}^{2}}\right)}^{2}{\left(\frac{n_{0}w_{0}^{2}}{{\mu^{\prime }}_{Y}\lambda_{0}K^{2}}\right)}^{-3/2}\\ +\frac{3\sqrt{\pi }}{4}{\left(\frac{n_{0}w_{0}^{2}}{{\mu^{\prime }}_{Y}\lambda_{0}K^{2}}\right)}^{-5/2} \end{array}\right) \end{array}\right]}^{-1}\\ & \cdot \left(\begin{array}{l} -\frac{15\mu_{0}n_{0}}{16{\mu^{\prime }}_{Y}\lambda_{0}K^{2}}\frac{\sqrt{\pi }}{{\left(\frac{n_{0}w_{0}^{2}}{{\mu^{\prime }}_{Y}\lambda_{0}K^{2}}\right)}^{7/2}}+\frac{45}{4}{\left(\frac{\mu_{Y}\lambda_{0}K}{w_{0}^{2}}\right)}^{2}\frac{\sqrt{\pi }}{{\left(\frac{n_{0}w_{0}^{2}}{{\mu^{\prime }}_{Y}\lambda_{0}K^{2}}\right)}^{5/2}}\\ +\left(\frac{15}{2}{\left(\frac{\mu_{Y}\lambda_{0}K}{w_{0}^{2}}\right)}^{4}-\frac{1}{2}\right)\frac{\sqrt{\pi }}{{\left(\frac{n_{0}w_{0}^{2}}{{\mu^{\prime }}_{Y}\lambda_{0}K^{2}}\right)}^{3/2}}+\left(\begin{array}{l} {\left(\frac{\mu_{Y}\lambda_{0}K}{w_{0}^{2}}\right)}^{2}\\ -\frac{\mu_{0}n_{0}}{2{\mu^{\prime }}_{Y}\lambda_{0}K^{2}}{\left(\frac{\mu_{Y}\lambda_{0}K}{w_{0}^{2}}\right)}^{6} \end{array}\right)\frac{\sqrt{\pi }}{{\left(\frac{n_{0}w_{0}^{2}}{{\mu^{\prime }}_{Y}\lambda_{0}K^{2}}\right)}^{1/2}} \end{array}\right) \end{array}\;$$

## 3. Experimental Study

In this section, the developed experimental system and schedule will be presented in detail. The experimental system consists of three major devices: pretension device, load device, and data collection device. Then, the experimental schedule has three major items: samples, load program, and measurement parameters. The experimental study is aimed at validating the theoretical model proposed.

### 3.1. Experimental System

#### 3.1.1. Experimental Pretension Device

**Figure 4:**
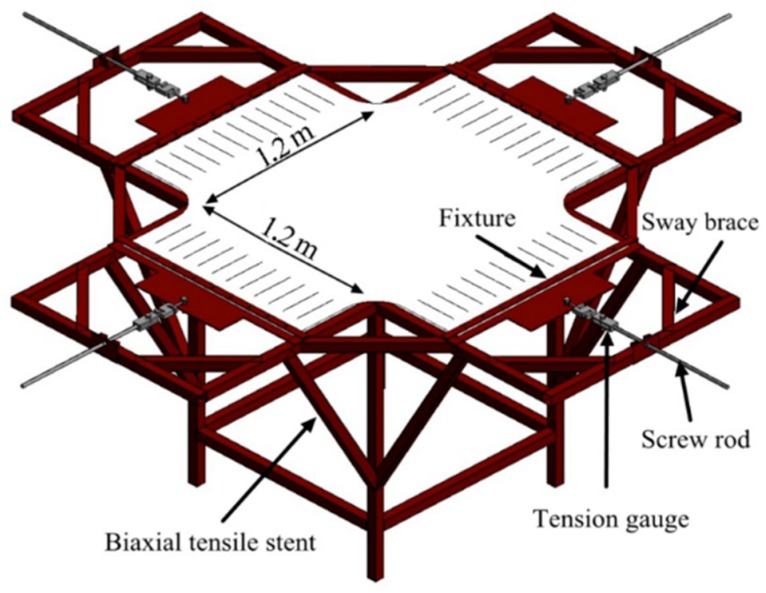
Illustration of pretension device.

As is shown in Figure 4, rectangular membrane is tensioned uniformly by the pretension device [27]. The pretension force is provided by the screw rod at each edge. For every fixture, 11 screw holes are laid uniformly and used to fix the membrane and transfer the pretension uniformly. The tension gauge connects the screw rod and fixture, which is applied to measure and control pretension force. 

#### 3.1.2. Load Device

**Figure 5:**
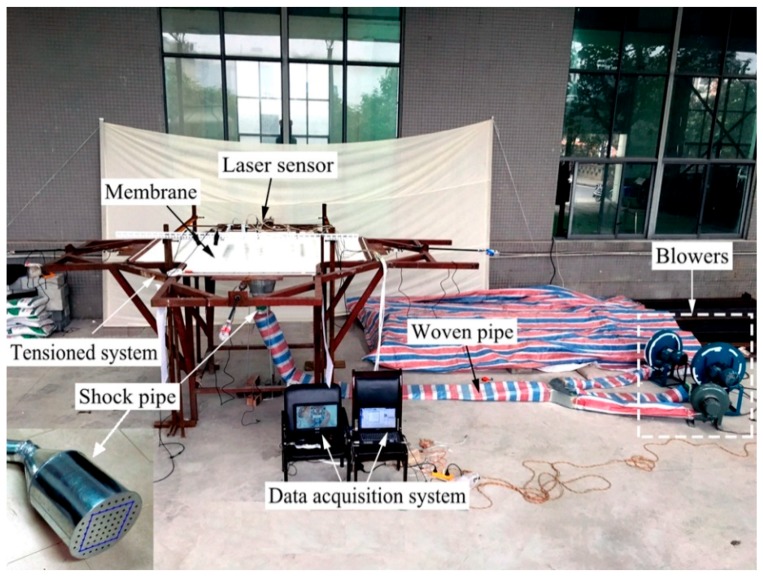
Photo of whole experimental system.

The pulse wind sourced from the developed load system is used to mimic the impact load. In order to realize the rectangular impact area, the holes arranged outside the rectangular area were blocked by tapes. The load system mainly consists of high pressure centrifugal blowers, woven pipe, and shock pipe, among others. The instantaneous characteristics of impact load can be achieved by instantly stepping on the woven pipe. The different velocity of wind can be realized using different high pressure centrifugal blowers. A photo of the whole experimental system is presented in Figure 5. The parameters of high pressure centrifugal blowers are presented in Table 1.

#### 3.1.3. Data Collection Device

**Figure 6:**
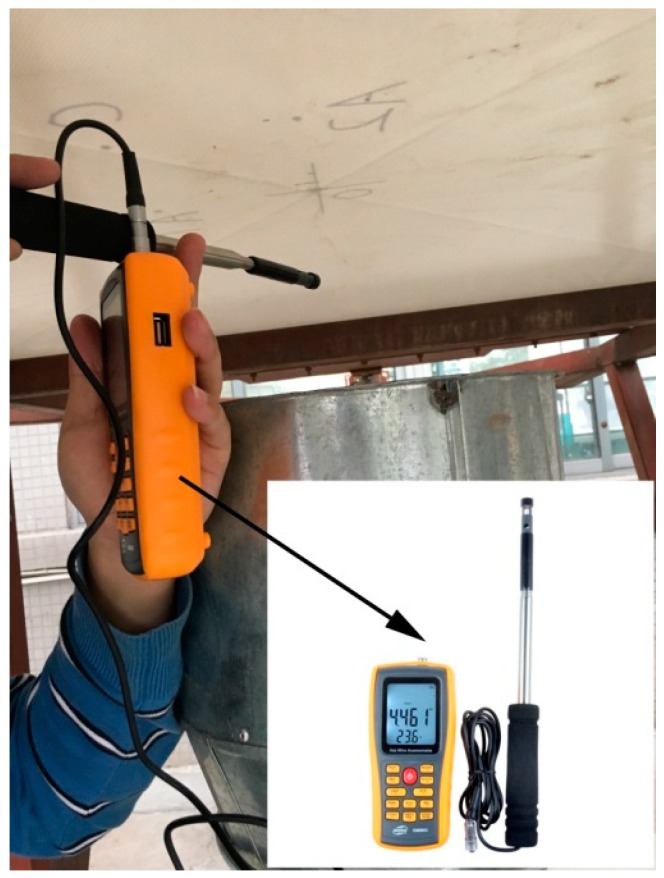
Digital velocity measurement.

The data collection includes the key parameters of pretension force, velocity of wind and displacement. The pretension force can be obtained by the digital pretension device. The velocity of wind can be measured by the digital velocity device shown in Figure 6, which has 3% precision and 0–30 m/s range, respectively. The transverse displacement of membrane can be measured and collected by the laser sensor, which possesses 0.1% precision and 2000 Hz sampling rate [27].

### 3.2. Experimental Schedule

#### 3.2.1. Experimental Samples

**Figure 7:**
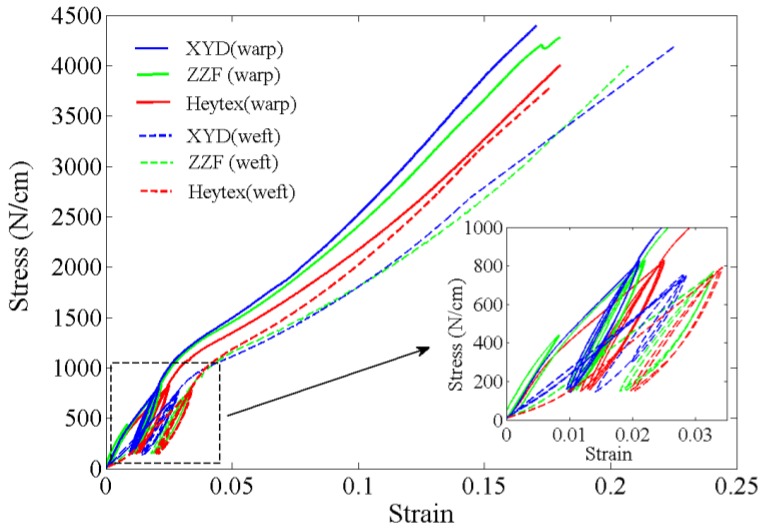
The stress-strain curves of membrane materials.

Three bands of membrane material including Heytex, ZZF and XYD, which have been applied widely in engineering field, are selected to analyze. The material properties are all tested and shown in Figure 7 [13]. In order to eliminate the effect of membrane material hysteresis and obtain the accurate elastic modulus results, the material test should be performed beginning with three load–unload cycles [28]. Furthermore, the key parameters can be extracted and presented in Table 2 [13]. The size of membrane sample is 1.2 m × 1.2 m. The experimental samples are all cut into stripes at each edge to realize the transmission of pretension force.

#### 3.2.2. Load Program

**Figure 8:**
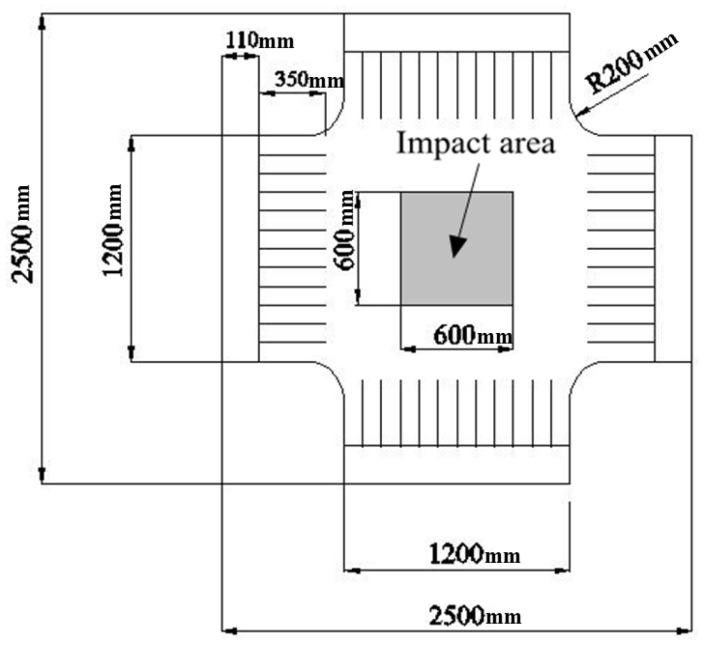
Illustration of experimental samples and impact area.

At the first step, the pretension force designed with 1000, 2000, 3000, 4000, and 5000 N, respectively, is applied towards each edge. Under each level of pretension force, the pulse wind induced by high-pressure blower is imposed into impact area illustrated in Figure 8. According to the theory of the Monte Carlo method [29], the experiment for each case should be performed 300 times repeatedly.

#### 3.2.3. Measurement Parameters

**Figure 9:**
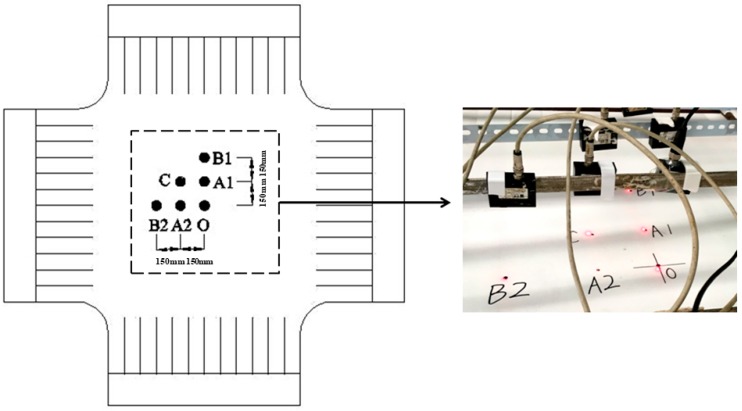
Distribution of measurement points.

**Figure 10:**
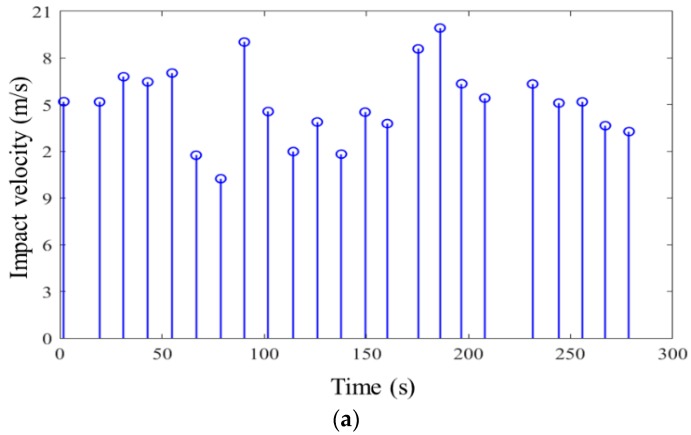

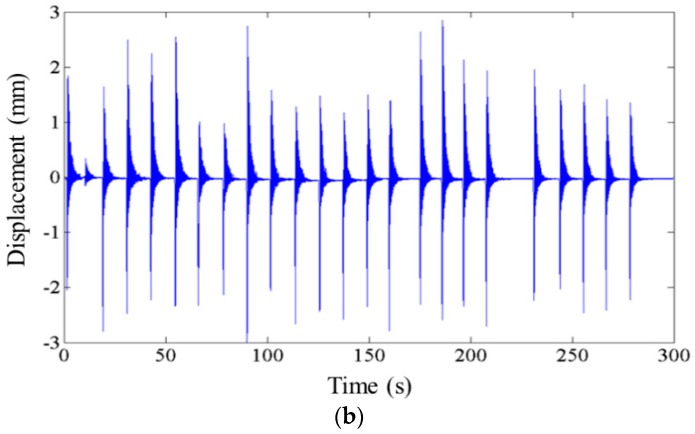
Some experimental data samples at Point A2. (**a**) Impact velocity with respect to time; (**b**) displacement with respect to time.

**Figure 11:**
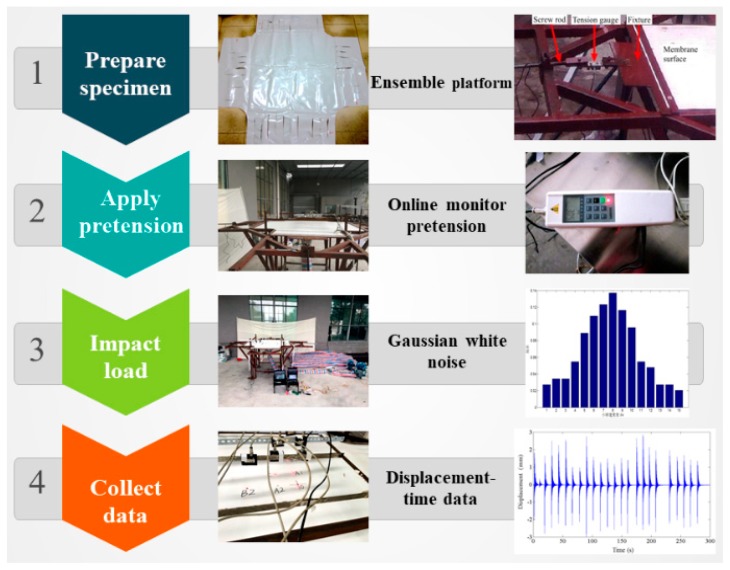
Flow chart of the experimental program.

The measurement parameters consist of wind velocity and displacement of membrane. A set of velocity value of impact wind load could be obtained and formed the data basis to perform the statistical analysis. As is shown in Figure 9, the measurement points are located at Point O, Point A1, Point A2, Point B1, Point B2, and Point C, respectively. The displacement-time curves at specific points could be collected and used to analyze the stochastic response characteristics of membrane system. Some experimental samples of random impact velocity and the corresponding displacement with respect to time at Point B1 are depicted in Figure 10. Finally, the developed experimental system and program are summarized as the brief flow chart shown in Figure 11. 

## 4. Validation of Theoretical Model

### 4.1. Impact Load

**Figure 12:**
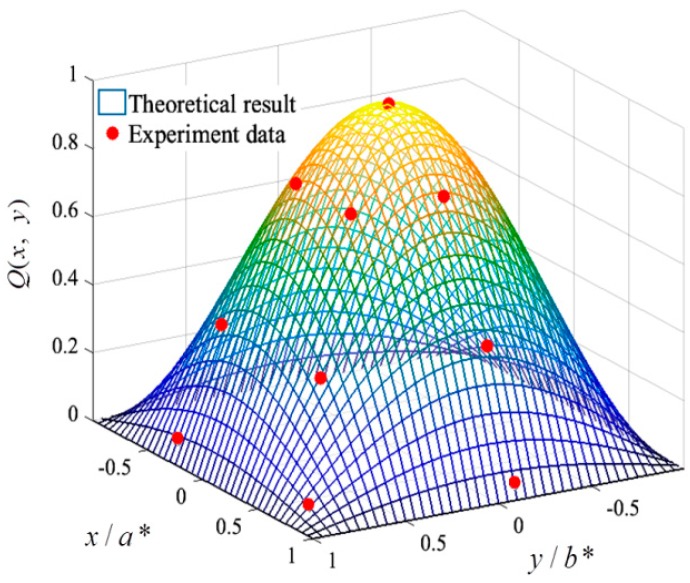
Validation of spatial distribution of impact load.

**Figure 13:**
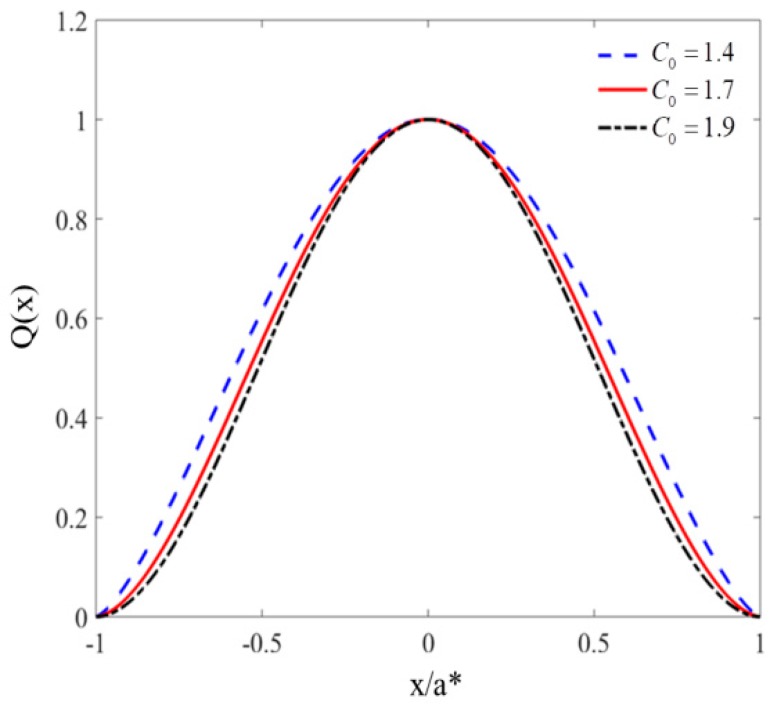
One-dimensional spatial distribution shape of impact load.

The spatial distribution results obtained from theory and experiment are given and compared in Figure 12. The curve surface presents the theoretical results obtained from Equation (2), and the red point shows the experimental results acquired from the experiment. It can be observed that the experimental points are almost located around the theoretical surface, which indicates the reliability of the theoretical model. In addition, because the load and membrane are symmetric, the spatial shape parameters $C_{1}$ and $C_{2}$ are equal. After interpolation analysis of experimental data, the one-dimensional spatial distribution shape could be obtained and is shown in Figure 13. Furthermore, the spatial shape parameter $C_{1}$ and $C_{2}$ can be extracted as follows: 1.9 (maximal wind), 1.7 (medium wind), and 1.4 (minimum wind).

**Figure 14:**
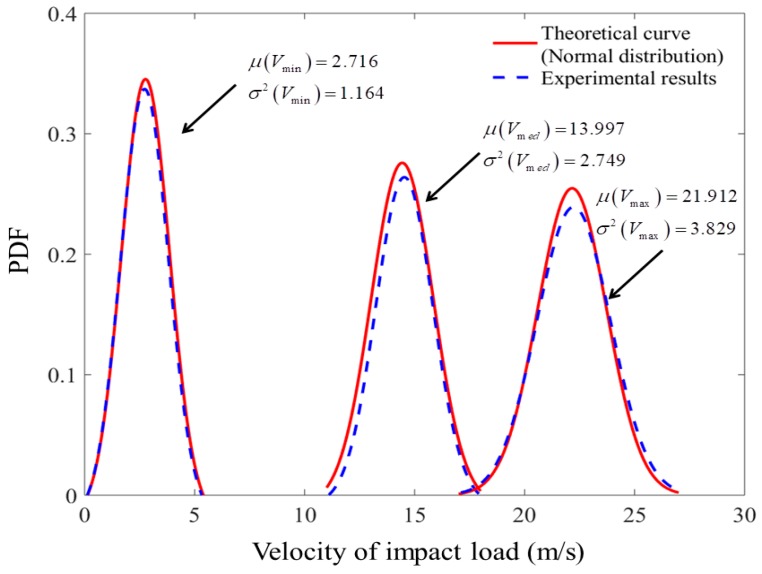
Comparison of PDF results of random wind velocity.

The PDF results of velocity of impact wind load at Point O obtained from experiment and their fitted theoretical PDF curve are plotted in Figure 14. It can be observed that the theoretical and experimental solutions are well matched. Based on the percentage error analysis listed in Table 3, the error varies in a very limited manner with the maximal error of 4.60%. Furthermore, the PDF of velocity of impact wind load basically follows the Gaussian distribution, which validates the assumption of the Gaussian distribution for random impact load. The method of least squares was applied to identify the key parameters, including mean and variance value, which are listed in Table 3 for the three cases of random impact load induced by different power blowers. The set of parameters reveal that the variance of velocity will increase with their mean value increasing.

### 4.2. Stochastic Vibration Characteristic

The monte carlo simulation (MCS) method is believed to be one of the most effective tools to predict the probabilistic characteristics of a system. A set of experimental samples can be used to calculate the simulated solution of stochastic vibration. The accuracy of results based on the MCS depends on the number of the statistical samples. In order to make the statistical error within 5%, the number of experimental samples is designed to be 300 [29].

**Figure 15:**
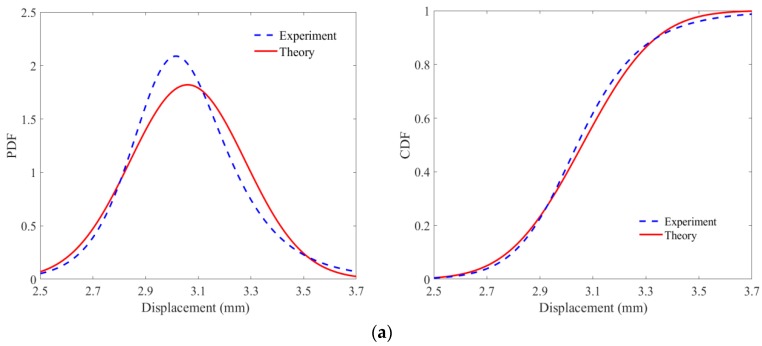

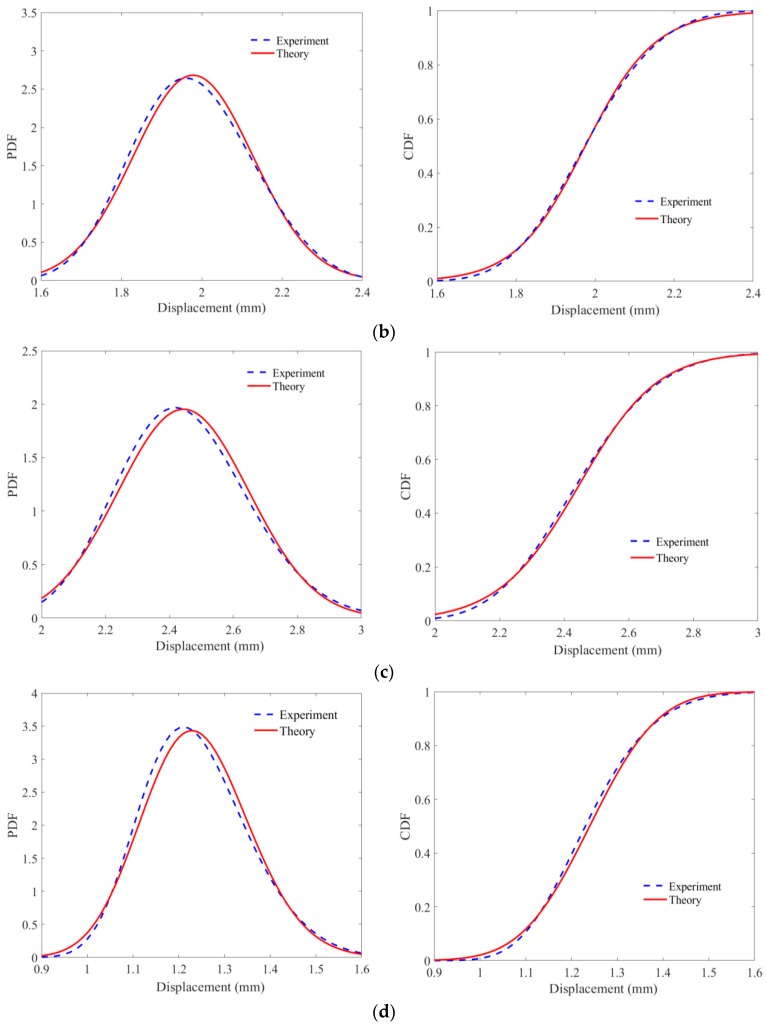

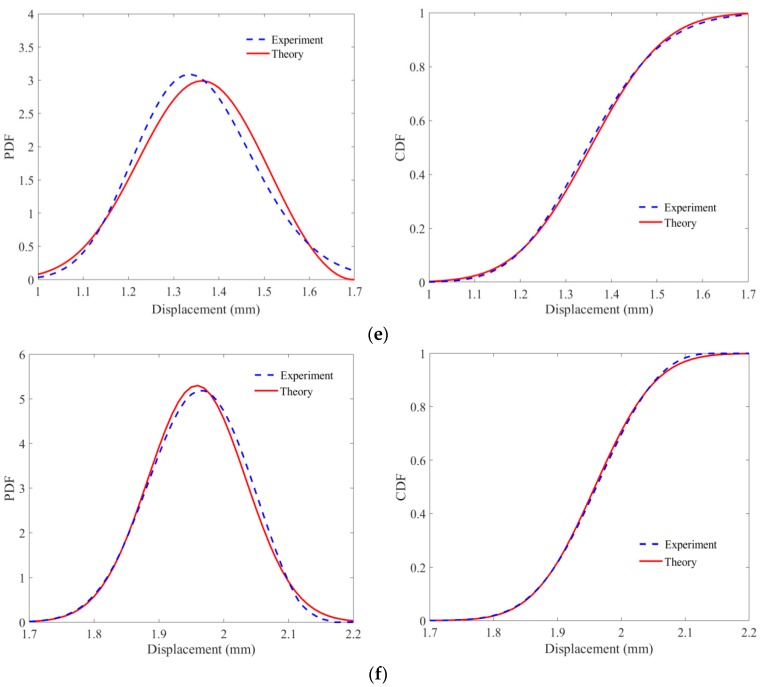
PDF and CDF results of displacement at specific positions: (**a**) Point O, (**b**) Point A1, (**c**) Point A2, (**d**) Point B1, (**e**) Point B2, and (**f**) Point C.

Figure 15 illustrate the PDF and CDF results of displacement at Point O, respectively, in which case the mean value of velocity of impact wind load is 13.997 m/s and the pretension force is 1000 N. It can be seen that the theoretical results calculated by the FPK method are almost consistent with the experimental results obtained by the MCS method. For the position with the most dominant dynamic response, namely Point O, the mean values of displacement obtained by the theoretical model and experiment are 3.035 and 2.983 mm, respectively, with the error of 1.743%. The discrepancy occurs partly from the assumption of ignoring the reflecting velocity of random impact load.

Besides this, another interesting phenomenon should be noted that when the strong stochastic vibration with larger deformation and more nonlinearity takes place in the central region, such as Point O, the experimental PDF curve of displacement will prove non-Gaussian distribution, which may indicate the function of membrane as the nonlinear filter system. However, when the energy of random impact load is limited to only being able to induce weak stochastic vibration occurring at the edge region, such as Point A1, Point A2, and Point C, the PDF curve of displacement will approximate the Gaussian distribution, which may suggest the function of membrane as the linear filter system.

Table 4 and Table 5 present the mean value and variance value of displacement at different measurement locations. It is easily found that the discrepancy between the theoretical result and experimental result are very limited, with the largest error of 2.035% for mean value and 6.452% for variance value. When the mean value of displacement decreases from central region (Point O) to edge region (Point B1 and B2), the variance value will reduce as well, which reveals that the random characteristics of random vibration are reducing.

## 5. Results and Discussions

The displacement of membrane subjected to impact load may exceed deformation capacity, which will further lead to the crack and tearing of membrane. Thus, the displacement is regarded as the vital parameter for the stochastic vibration analysis and dynamic design [30]. In this section, the effects of variables, including pretension force, velocity of impact load, and membrane material, on the stochastic results of displacement are discussed in detail.

### 5.1. Effect of Pretension Force

#### 5.1.1. Effect of Pretension Force on Mean Value of Displacement

**Figure 16:**
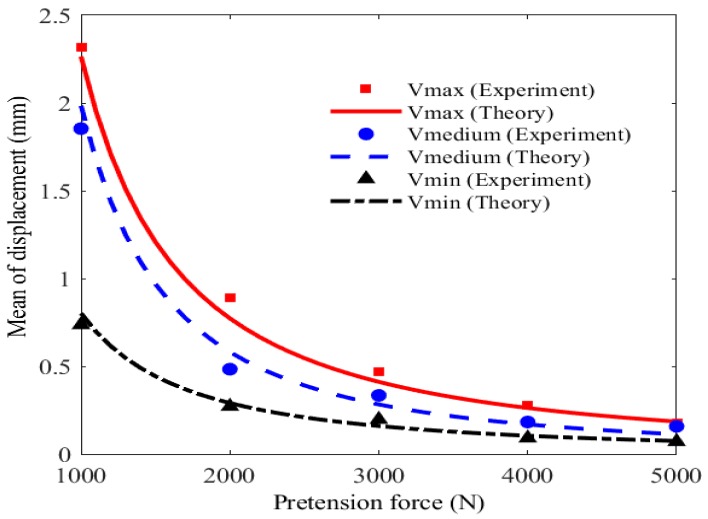
Mean value of displacement versus pretension force.

The effect of pretension force on the mean value of displacement at Point A1 is illustrated and discussed in Figure 16. For the case that the mean value of velocity of impact wind load is 13.997 m/s and the pretension force is 1000 N, the theoretical result obtained by the FPK method is approximate to experimental data acquired from the MCS method, with the error of 0.605%. From the figure, the mean value of displacement declines 31% averagely and nonlinearly with the pretension force increasing from 1000 to 3000 N. However, this trend becomes stable with the declination of 6% when the pretension force varies from 3000 to 5000 N. The phenomenon indicates that the pretension force varying within 3000 N has obvious effect on the stiffness of membrane.

#### 5.1.2. Effect of Pretension Force on Variance Value of Displacement

**Figure 17:**
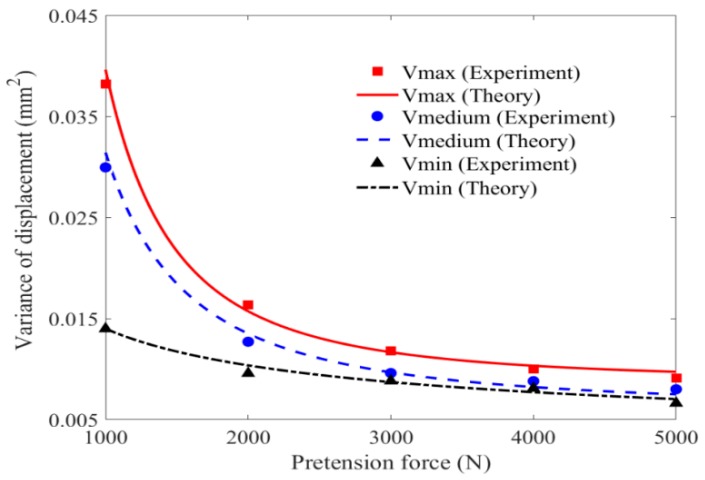
Variance value of displacement versus pretension force.

The effect of pretension force on the variance value of displacement is illustrated and discussed in Figure 17. It can be seen the experimental values are distributed around the theoretical curves. For the case that the mean value of velocity of impact wind load is 13.997 m/s and the pretension force is 1000 N, the theoretical result calculated by the FPK method is consistent with the experimental value, with the error of 5.969%. Similar to the rule of mean value, the variance reduces obviously and nonlinearly with the pretension force increasing within 3000 N. When pretension force is over 3000 N, the influence tends to be gentle. This suggests that the displacement within 3000 N is more dispersed and worthy of consideration carefully in design.

### 5.2. Effect of Velocity of Impact Load

#### 5.2.1. Effect of Velocity of Impact Load on Mean Value of Displacement

**Figure 18:**
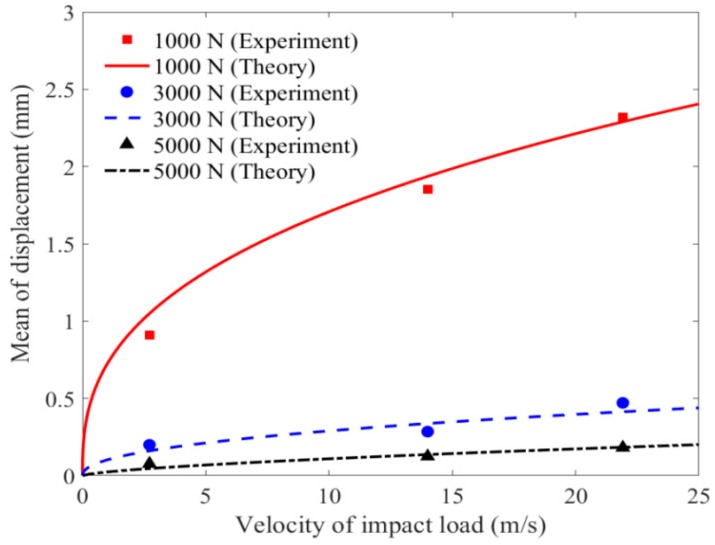
Mean value of displacement versus velocity of impact wind.

The effect of velocity of impact load on the mean value of displacement at Point A1 is presented and discussed in Figure 18. It can be found that there is a nonlinear trend in the displacement–velocity relationship with pretension force, particularly for the cases of pretension force with 1000 N. Moreover, the slope tends to reduce along with the increase of velocity of impact load, revealing the hard-spring characteristics of membrane system. Another interesting phenomenon can be observed in that the displacement–velocity curve is approximate to linear relationship when the velocity of impact load varies within 3 m/s. Then, the displacement starts to rise nonlinearly with the velocity of impact load increasing over 3 m/s. This phenomenon indicates that membrane subjected to larger velocity of impact load shifts the stochastic vibration characteristic from linearity to hardening nonlinearity type, and the feature of hardening nonlinearity is more pronounced with lower pretention force.

#### 5.2.2. Effect of Velocity of Impact Load on Variance Value of Displacement

**Figure 19:**
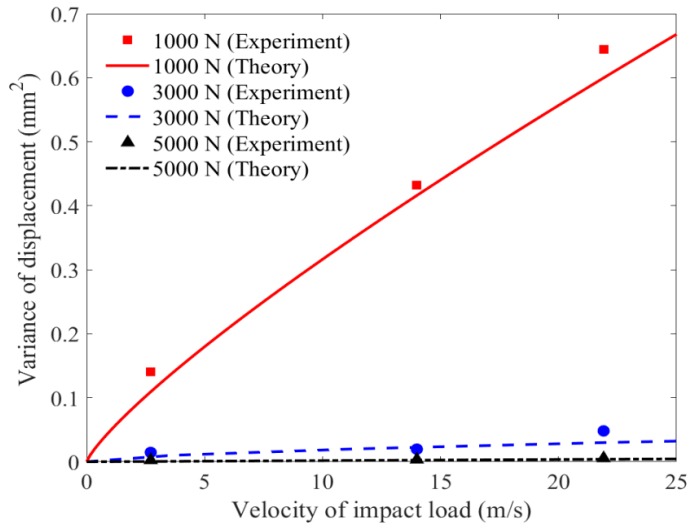
Variance value of displacement versus velocity of impact wind.

The effect of velocity of impact load on the variance value of displacement at Point A1 is shown and discussed in Figure 19. Similar to the effect of velocity of impact load on mean value of displacement, the variance of displacement increase significantly with the rise of velocity of impact load, indicating that the statistical results of stochastic vibration spread out more when membrane is subjected to larger velocity of impact load. In addition, the change of slope does not vary obviously as the condition of mean results show in Figure 18, which means that the rise of variance due to the increase of velocity of impact load is faster than the case of the mean results, especially for the case with the lower level of pretension force.

### 5.3. Effect of Membrane Material

#### 5.3.1. Effect of Membrane Material on Mean Value of Displacement

**Figure 20:**
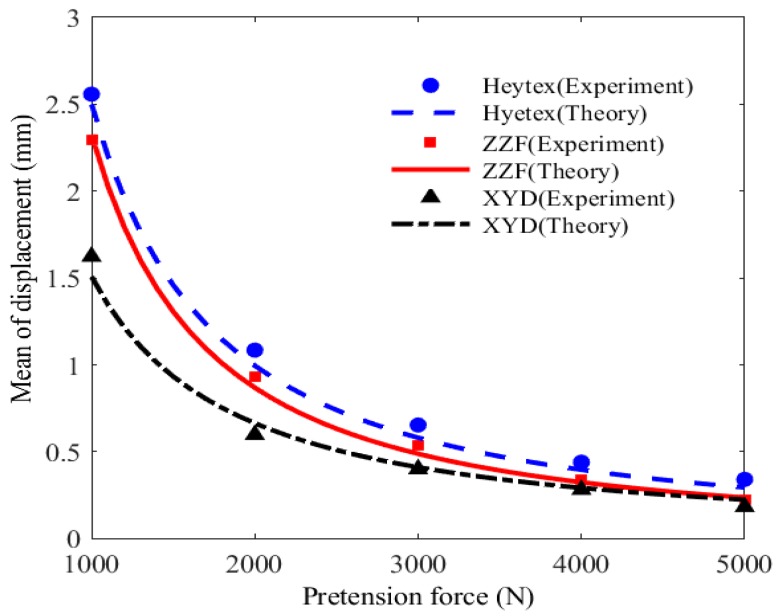
Mean value of displacement versus membrane material.

The effect of membrane material on the mean value of displacement at Point A2 is depicted and discussed in Figure 20. This case is that the membrane is subjected to the impact load with mean velocity of 13.997 m/s. For Heytex membrane material, the mean value of displacement is the maximal. In contrast, the result obtained from XYD membrane material is the minimal. It could be explained by the fact that the elastic modulus of Heytex membrane material is the minimal, which results in the low structural stiffness. Thus, Heytex membrane is more prone to vibrate stochastically subjected to the impact load.

#### 5.3.2. Effect of Membrane Material on Variance Value of Displacement

**Figure 21:**
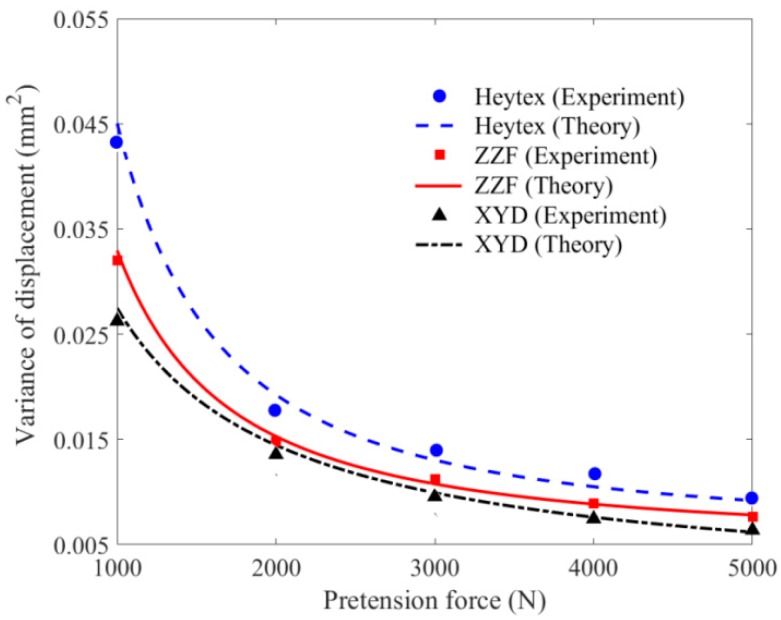
Variance value of displacement versus membrane material.

The effect of membrane material on the variance value of displacement at Point A2 is illustrated and discussed in Figure 21. Unlike the effect of pretension force and velocity of wind, the effect of membrane material on variance value of displacement is obvious within 1000 N. It is considered that the difference of elastic modulus for these three types of membrane material is not large, only with about 4.8% discrepancy. Therefore, the effect of membrane material on the variance result can be ignored when the pretension force exceeds 1000 N.

## 6. Conclusions

In this paper, the stochastic vibration problem of orthotropic membrane subjected to random impact load is investigated theoretically and experimentally. In the theoretical part, the model of random impact load is initially developed as white Gaussian noise input with respect to the membrane system based on the stochastic pulse theory. Then, the FPK governing motion equations for geometrically nonlinear membrane are derived and solved based on Von Karman theory and FPK method. Consequently, the probabilistic and statistical results can be identified. Afterwards, the theoretical model is validated by experimental study. Furthermore, the parameter discussions including pretension force, velocity of impact load, and membrane material on stochastic vibration behavior are performed. The main conclusions can be summarized as follows:

- The theoretical model proposed can predict the stochastic dynamic characteristics of the membrane accurately subjected to random impact load;
- When the strong stochastic vibration with obvious nonlinearity occurs, membrane will function as the nonlinear filter system, with the PDF results of dynamic response prone to approximate the Rayleigh Distribution. However, when the stochastic vibration proves weak, membrane will function as the linear filter system, with the corresponding PDF results more likely to follow the Gaussian distribution.
- The developed experimental system and program paves a way to study the stochastic vibration problem of membrane subjected to random impact load;
- The mean and variance value declines nonlinearly with pretension force and elastic modulus increasing. However, it will rise with velocity of impact load increasing. Furthermore, pretension force affects stochastic vibration results most dominantly among different variables.

## Figures and Tables

**Table 1:** Parameters of the high-pressure blower.

Blower Type	Power (W)	Flow (m^3^/h)	Pressure (Pa)	Rotational Speed (r/s)
CZR-CZT	1100	1140	1080	2800/60
Y90L-2	2200	1191	2562	2840/60
Y100L-2	3000	1704	3253	2880/60

**Table 2:** The brief introduction of membrane material.

Type	Density (kg/m²)	Thickness (mm)	Poisson Ratio (Warp/Weft)	Elastic Modulus (Warp/Weft) (MPa)	Tensile Strength (Warp/Weft) (N/cm)
Hextex	0.95	0.8	0.3/0.4	1520/1290	4000/3800/5
ZZF	0.95	0.8	0.3/0.4	1590/1360	4300/4000/5
XYD	0.95	0.8	0.3/0.4	1720/1490	4400/4200/5

**Table 3:** Comparison of mean and variance value of random wind velocity.

Wind Type	Mean Value			Variance Value		
	Theory (m/s)	Experiment (m/s)	Error (%)	Theory (m^2^/s^2^)	Experiment (m^2^/s^2^)	Error (%)
Minimum wind	2.716	2.652	2.41	1.164	1.142	1.93
Medium wind	13.997	14.187	−1.34	2.749	2.628	4.60
Maximal wind	21.912	22.017	−0.48	3.829	3.874	−1.16

**Table 4:** Comparison of mean value of displacement at measurement points.

Position	Theory (mm)	Experiment (mm)	Error (%)
Point O	3.035	2.983	1.743
Point A1	1.994	1.982	0.605
Point A2	2.457	2.408	2.035
Point B1	1.248	1.224	1.961
Point B2	1.368	1.342	1.937
Point C	1.952	1.974	1.114

**Table 5:** Comparison of variance value of displacement at measurement points.

Position	Theory (mm^2^)	Experiment (mm^2^)	Error (%)
Point O	0.033	0.031	6.452
Point A1	0.031	0.029	5.969
Point A2	0.032	0.030	5.864
Point B1	0.018	0.017	3.053
Point B2	0.024	0.023	4.013
Point C	0.030	0.028	6.078
